# A Comprehensive Benchmark Database of Per- and Polyfluoroalkyl Substance Properties from Quantum Mechanical Methods

**DOI:** 10.1021/acs.jcim.5c03092

**Published:** 2026-04-29

**Authors:** Melissa Marciesky, Scott Simpson, John Keith, Carla Ng

**Affiliations:** † Department of Chemical and Petroleum Engineering, 6614 University of Pittsburgh, Pittsburgh, Pennsylvania 15260, United States; ‡ Department of Chemistry, St. Bonaventure University, Buffalo, New York 14778, United States; § Department of Civil and Environmental Engineering, University of Pittsburgh, Pittsburgh, Pennsylvania 15260, United States

## Abstract

Per- and polyfluoroalkyl substances (PFAS) remain challenging for quantum mechanical (QM) modeling due to their atypical electronic structures, large number of compounds, and limited experimental data for validation. To address this, we constructed a comprehensive benchmarking database that evaluates the performance of a broad range of QM methods. The database benchmarks thermal properties (BDEs) and electronic properties (dipole moments, electron affinities, ionization potentials), validated against experimental data where available and against high-level theoretical references. All data are released through the open and extensible PFAS_Database on GitHub, which is designed to grow over time, both in number of PFAS and inclusion of newly identified top-performing methods, providing a foundation for more reliable modeling and remediation strategies.

## Graphical abstract

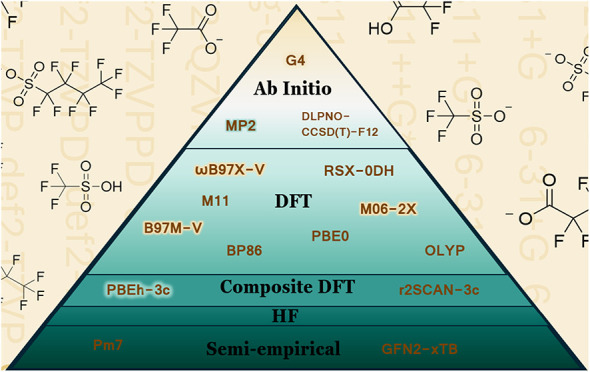

## 1 Introduction

Per- and polyfluoroalkyl substances (PFAS) are synthetic chemicals with fully (perfluorinated) or partially (polyfluorinated) carbon chains.^1^ One study identified more than 60 PFAS use categories and over 200 specific applications, including apparel, food production, fire-fighting foam, musical instruments, glass, and leather.^2^ Their widespread use and persistence have led to global contamination.^3–5^ In the United States, PFAS are detectable in the blood of nearly the entire population.^6,7^ Exposure has been linked to thyroid disease, insulin dysregulation, liver disease, and adverse reproductive outcomes.^8^ The exceptional stability of the C–F bond (105.4 kcal/mol^9^) makes PFAS resistant to degradation and, combined with their chemical diversity, complicates remediation. A hallmark of PFAS is that their high surface activity and persistence make them distinct from other compound classes, yet the majority of the thousands PFAS in use have not been experimentally evaluated.

Quantum mechanical (QM) methods offer a way to understand and predict PFAS properties, but their accuracy and cost for highly fluorinated systems remain uncertain. Chemical accuracy is typically defined within ± 1 kcal mol^–1^ (±4.184 kJ mol^–1^), a standard difficult to achieve consistently. Among *ab initio* methods, Coupled Cluster with Single, Double, and perturbative Triple excitations [CCSD­(T)] ^10–12^ is considered the “gold standard” for small molecules, but its O­(*N* ^7^) scaling limits practicality.^13^ Lower-cost *ab initio* methods, such as Møller–Plesset perturbation (MP) theory^14^ and composite schemes, reduce expense while retaining much of the accuracy, yet remain computationally intensive for large PFAS data sets.

Kohn–Sham Density Functional Theory (DFT)^15^ is the most widely used approach in PFAS studies, and is expected to offer a balance between cost and accuracy. Unlike *ab initio* methods, DFT is not systematically improvable. Perdew’s “Jacob’s Ladder”^16^ is often used to rank the accuracy of DFT functionals. Although the ladder conceptually ascends toward chemical accuracy, no single functional is universally reliable for all molecules and properties.^17^ PFAS, with their unusual electronic structures and diverse head groups, present a stringent test. Benchmarking DFT functionals within this framework is therefore essential for identifying methods that yield reliable PFAS results.

The electronic properties of PFAS influence their physicochemical behavior and reactivity. Dipole moments, which describe electron density and molecular polarity, help characterize intra- and intermolecular interactions.^18^ In PFAS, dipole–dipole interactions govern F–F contacts,^19^ whereas local C–F bond dipoles cancel, yielding a nonpolar, hydrophobic fluoroalkyl chain that lowers fluorosurfactant surface tension.^20,21^ Vyas and co-workers used DFT calculations to study perfluorocarboxylic acetate reduction, finding that different methods produced inconsistent geometries and energies, but ωB97X-D delivered the most reliable results, reproducing reduction potentials of perfluorooctanoate radical (PFOA^•^) and perfluorooctanesulfonate radical (PFOS^•^).^22^ These results highlight both the promise and potential variability of DFT for PFAS, underscoring the need for systematic benchmarks.

Thermodynamic properties of PFAS have also been investigated through reaction-scheme approaches that enable systematic error cancellation. Multiple isogyric schemes^23–24^ have been applied with the ANL0 method to study small C/F/O/H molecules benchmarked against the Active Thermochemical Tables (ATcT).^26–28^ Owing to its performance, the ANL0 database has been instrumental in advancing PFAS thermochemistry. Ram, Westmoreland, and co-workers used the same isogyric scheme, combining reference species from ATcT and ANL0, to calculate enthalpies of formation for open- and closed-shell perfluorinated carboxylic and sulfonic acids. Using G4 as a reference method benchmarked against ANL0 and ATcT data,^29^ they evaluated species up to four carbons in length, considered short-chain PFAS (perfluoroalkanesulfonic acids (PFSAs) and perfluoroalkanoic acids (PFCAs) with fewer than six or seven carbons, respectively).^30^ These G4 results were then used to assess the performance of other methods and basis sets (B3LYP, ωB97XD, M062-X, M06–2X-d3(0), and CBS-QB3).^31,32^ Abeywardane and Goldsmith employed a connectivity-based hierarchy (CBH) approach for closed-shell carboxylate PFAS, combining systematic error cancellation with dual-level composite protocols (high-level single-point CCSD­(T) or DLPNO–CCSD­(T) on DFT-optimized geometries).^33^ Similarly, bond dissociation energies (BDEs) of anionic PFAS have been reported using B3LYP/6–311+G­(2d,2p) with Grimme’s empirical dispersion correction and Becke–Johnson damping,^34–38^ along with Truhlar’s SMD solvation model^39^ to implicitly simulate aqueous environments, though such studies remain limited in scope.^40^

Given the vast number of PFAS and wide range of QM methods available, identifying sources of error is critical, especially in the absence of experimental data. Experimental thermochemical data for PFAS remain scarce and are largely restricted to small molecules with similar atomic environments. Although high-level composite and coupled cluster methods have successfully predicted enthalpies of formation, they are computationally impractical for large PFAS data sets and have not been validated for anionic species or electronic properties. Comprehensive benchmarking of electronic and thermodynamic properties can pinpoint method-specific errors, enabling selection of approaches that balance accuracy and efficiency for large-scale data sets.

This work aims to validate computationally efficient and reliable methods for PFAS simulation while clarifying trade-offs between accuracy and cost. We examine neutral, radical, anionic, and anionic radical PFAS with both carboxylate and sulfonate head groups. Key electronic and thermodynamic properties including dipole moments, ionization potentials, electron affinities, and bond dissociation energies are evaluated across a wide range of QM methods. Ultimately, this study establishes a framework for accurate and efficient PFAS property prediction.

## 2 Methods

### 2.1 PFAS Selection and Benchmarking Sets

An initial benchmark set of 50 PFAS-like small molecules was compiled, each with at least one experimentally available property: bond dissociation energy (BDE), dipole moment, electron affinity, or ionization energy. These benchmarking data were taken from the NIST Chemistry WebBook,^41^ the ATcT database, and the Hait and Head-Gordon molecular data set calculated using CCSD­(T)/complete basis set (CBS) limit.^42^ This set is hereafter referred to as Data Set 1.

In the absence of extensive experimental data for many PFAS, most available reference values correspond to small molecules with similar local atomic environments (e.g., HF, CH, CF). To bridge this gap and enable benchmarking on short-chain PFAS, smaller analogues were systematically constructed to cover perfluoroalkyl carboxylic acids (PFCAs) of the form CO_2_–(CF_2_)_*n*_–CF_3_ and perfluoroalkyl sulfonic acids (PFSAs) of the form SO_3_–(CF_2_)_*n*_–CF_3_ with *n* = 0–3. This data set of PFAS and PFAS-like compounds, hereafter referred to as Data Set 2, includes protonated and deprotonated forms of perfluorobutanoic acid (PFBA), perfluorobutanesulfonic acid (PFBS), hexafluoropropylene oxide dimer acid (HFPO–DA, Gen-X), 4:2 fluorotelomer carboxylic acid (4:2 FTCA), and 4:2 fluorotelomer sulfonic acid (4:2 FTS), together with radicals formed from C–C bond cleavage and fluorine abstraction. It spans four major structural subclasses (PFCAs, PFSAs, perfluoroalkyl ether carboxylic acids (PFECAs), and fluorotelomer substances) and substantially increases the number of available benchmarking points for computationally expensive approaches. In total, composite-level data are provided for 287 distinct molecules (neutral and anionic), and all benchmarked calculations were performed in the gas phase for both protonated and deprotonated forms.

Each property-specific benchmarking data set was constructed from the PFAS and PFAS-like structures described above but varies in the number of data points due to factors such as convergence behavior, availability of experimental references, choice of validation method, self-consistent field (SCF) energy convergence issues for some radicals, and computational cost. High-level composite methods and large-basis correlated calculations (e.g., CCSD/aug-cc-pVTZ) were prohibitively expensive for the full set; however, their performance for feasible compounds is included in the database for completeness. For each property, the data set includes the largest subset of PFAS for which reliable results were obtained, resulting in at least 60 data points for protonated species and 39 for deprotonated species per property. Note that the number of data points varies per molecule and property being evaluated. For instance, BDEs may have multiple entries per molecule, whereas dipole moments have one per molecule. The number of data points is denoted as *n* = *x* at the bottom of each graph for reference. For the exact molecules used for each property, refer to the PFAS_Database. This approach ensures that each data set maintains statistical reliability while maximizing coverage across molecular types. Complete details for each data set, including molecular structures and reference values, are provided in the Supporting Information and the PFAS_Database.

### 2.2 Benchmarking Computational Methods

We selected 47 methods for this study, representing a diverse range of semiempirical, DFT, MP2, composite, and coupled-cluster approaches. For brevity, Table 1 lists all methods explored in this work. Calculations were performed using both ORCA 6.0.1 ^43^ and Gaussian 16-A.03.^44^ ORCA was used for DLPNO–CCSD­(T)^45,46^ and all its variations, DSDBLYP,^47^ PWLDA,^48^ VWN5,^38^ OLYP,^50^ O3LYP,^38^ revPBE0,^52^ TPSSh,^53^ PW6B95,^54^ SCANfunc,^55^ ωB97X-V,^56^ RSX-0DH,^57^ TPSS,^53^ MN12SX,^58^ ωB97X-2,^59^ ωB97X-D3,^59^ RI-B2PLYP-D3BJ,^60^ ωB97M-V,^61^ B97-D,^62^ B97M-V,^63^ PBEh-3c,^64^ B3LYP-D3BJ,^34–36^ r2SCAN-3c,^65^ and GFN2-xTB.^66^ The semiempirical method GFN2-xTB was executed using the xtb code implemented through ORCA, while all remaining methods were performed in Gaussian 16. Basis sets were chosen from the Karlsruhe Def2 series (Def2-TZVPD) unless otherwise noted. The Karlsruhe basis family was implemented in Gaussian using the Gen keyword with definitions obtained from the Basis Set Exchange. Multilevel methods are expressed in the format: (electronic energy method)//(geometry, ZPE, and thermodynamic correction method). For example, CCSD­(T)/aug-cc-pVTZ//MP2/aug-cc-pVTZ indicates that the electronic energy was obtained at the CCSD­(T) level, while the geometry and thermodynamic quantities were derived at the MP2 level. *T*_1_ diagnostics were computed for molecules treated with coupled-cluster theory as a reliability metric. Values below 0.02 for main-group species and below 0.03 for radicals are normally reliable.^67,68^ Molecules exceeding these thresholds are better described using multireference methods. Spin contamination was evaluated for all open-shell species. Spin contamination has been shown to increase errors in DFT-based reaction energies.^69^ It is considered severe when greater than 0.1 ^70^ or 10%.^71^ Accordingly, this study adopted a 0.1 threshold for open-shell molecules (Δ⟨*Ŝ* ^2^⟩, eq 1). Molecules and methods exhibiting significant spin contamination are noted within the database. All thermodynamic calculations were also checked for large (|ν| ≥ 100 cm^–1^) imaginary frequencies. Any result failing key quality-control criteria (*T*_1_ threshold, spin contamination, or imaginary frequency) was excluded on a per-molecule basis from statistical evaluations. Methods with frequent exclusions are explicitly discussed.

1 $$\Delta \left\langle {Ŝ}^{2}\right\rangle =\left\langle {Ŝ}^{2}\right\rangle -S_{z}\left(S_{z}+1\right)$$

**Table 1:** Methods Benchmarked in This Study Using PFAS Bond Dissociation Energies, Dipole Moments, Electron Affinities, and Ionization Potentials

Ab Initio	*Coupled-cluster*	CCSD,^72^ CCSD(T),^10^ DLPNO–CCSD(T),^45,46^ DLPNO–CCSD(T)-F12 ^74^
	*Composite*	CBS-QB3,^75^ CBS-APNO,^76^ W1BD,^77^ W1RO,^78^ G3,^79^ G4 ^80^
	*M*ø*ller–Plesset perturbation theory*	MP2 ^81^
		
**DFT**	Double Hybrid Meta GGA	
	*Double Hybrid GGA*	DSD-BLYP,^47^ B2PLYP with RI,^60^ RSX-0DH,^57^ WB97X-2 ^200^
	*Range Separated Hybrid Meta GGA*	ωB97M-V,^61^ M11,^82^ ωB97X-V,^56^ MN12SX^58^
	*Range Separated Hybrid GGA*	ωB97X-D3,^83^ CAM-B3LYP^84^
	*Hybrid Meta GGA*	M06,^85^ M06–2X,^85^ MN15,^86^ TPSSh,^53^ PW6B95 ^54^
	*Hybrid GGA*	B3LYP,^35–37^ PBE0,^91^ B3LYP-D3BJ,^62^ O3LYP,^51^ revPBE0 ^52^
	*Meta-GGA*	M06-L,^85^ M11-L,^92^ MN15-L,^86^ SCAN,^55^ B97M-V^63^
	*Generalized Gradient Approximation (GGA)*	PBE,^93^ BP86,^94,95^ BLYP,^36,94^ OLYP,^50^ B97-D^62^
	*Local-Density Approximation (LDA)*	VWN5,^38^ PWLDA^48^
		
	**Composite DFT**	r2SCAN-3c,^65^ PBEh-3c^64^
		
	**Hartree–Fock (HF)** ^96^	
		
	**Semiempirical**	PM7,^97^ GFN2-xTB^66^

Statistical analyses were performed for bond dissociation energy (BDE), ionization potential (IP), electron affinity (EA), and dipole moment data. For each method, the difference between the predicted value and the reference value was calculated as Φ_*i*_ = *E*_Validation_ – *E*_*i*_. In Data Set 1, *E*_Validation_ corresponds to experimental energy. In Data Set 2, it represents values from a validated computational method selected on the basis of performance in Data Set 1.

The mean signed error (MSE) (eq 2), mean unsigned error (MUE) (eq 3), and root-mean-square deviation (RMSD) (eq 4) were computed for bond dissociation energy, ionization energy, and electron affinity as follows, where *n* is the number of reference species available for that method. The maximum absolute deviation (Φ_max_) was also used as a metric to indicate the largest observed deviation for each method.

2 $$\mathrm{MSE}=\frac{\sum \Phi_{i}}{n}$$

3 $$\mathrm{MUE}=\frac{\sum \left|\Phi_{i}\right|}{n}$$

4 $$\mathrm{RMSD}=\sqrt{\frac{\sum {\left(\Phi_{i}\right)}^{2}}{n}}$$

### 2.3 Thermodynamic Properties

#### 2.3.1 Bond Dissociation Energy (BDE) and Basis Set Convergence

Homolytic BDEs were calculated using eq 5, where Δ*H* is the total change in enthalpy, *H*_AB_ is the enthalpy of the parent molecule, and *H*_A_ and *H*_B_ are the enthalpies of the resulting fragments. All thermodynamic corrections are calculated at 298.15 K and 1 atm, as obtained from the default settings in ORCA and Gaussian.

5 $$\Delta H=\left(H_{A}+H_{B}\right)-H_{\mathrm{AB}}$$

#### 2.3.2 Vibrational Scaling

Vibrational frequency corrections were applied using the quasi-rigid-rotor harmonic-oscillator (qRRHO) model with a unified cutoff of 100 cm^–1^.^98^ In ORCA, the default implementation employs the qRRHO scheme, whereas Gaussian applies the rigid-rotor harmonic-oscillator (RRHO) approximation by default. In this study, we found that deprotonated (anionic) PFAS often exhibit small-magnitude (|ν| ≤ 100 cm^–1^) imaginary frequencies in the gas phase. This behavior was observed across multiple methods and PFAS types in this work, suggesting that the low-frequency instabilities are numerical artifacts rather than physical phenomena. To ensure consistency across software packages, the qRRHO treatment was applied to all vibrational analyses, with any frequencies below 100 cm^–1^ replaced by a fixed value of 100 cm^–1^. Standard vibrational scaling factors, which are defined within the respective composite methods, were applied prior to the qRRHO correction. Any method–molecule combination exhibiting large imaginary frequencies (|ν| > 100 cm^–1^) was excluded from statistical analysis. However, these entries are still included in the PFAS_Database for transparency and completeness.

### 2.4 Electronic Properties

#### 2.4.1 Ionization Energy and Electron Affinity

Vertical ionization energy is found by subtracting the electronic energy of the positively charged species (*E*_el_^+^) in the ground-state optimized geometry of the neutral species from the electronic energy of the optimized neutral species (*E*_el_) (eq 6). Vertical electron affinity is found in a similar manner (eq 7); zero-point energy corrections are not included, consistent with the definition of vertical processes. The completed results for vertical ionization potentials and electron affinities are provided in the database, but will not be discussed.

6 $$IE_{vertical}=E_{el}^{+}-E_{el}$$

7 $$EA_{vertical}=E_{el}-E_{el}^{-}$$

Adiabatic ionization energy and electron affinity are computed in much of the same manner (eq 8 and eq 9). However, the charged species is optimized to its own minimum energy geometry. In addition, zero point energy (*E*_el+ZPE_) is also included in the energy total.

8 $$\mathrm{IE}=E_{\mathrm{el}+\mathrm{ZPE}}^{+}-E_{\mathrm{el}+\mathrm{ZPE}}$$

9 $$\mathrm{EA}=E_{\mathrm{el}+\mathrm{ZPE}}-E_{\mathrm{el}+\mathrm{ZPE}}^{-}$$

#### 2.4.2 Dipole Moment

Dipole moment magnitudes (μ) are taken from geometries optimized at the level of theory. For Gaussian files, the setting ‘‘density = current” is applied for DFT, HF, CCSD, and MP2. In ORCA, dipole moment is calculated using the ‘‘%elprop Dipole True” setting. Although dipole moments were collected for composite methods and are included in the database, they are excluded from benchmarking comparisons. This is because dipole moment reflects the density from an intermediate level of theory used within the composite scheme, rather than the full composite method itself. As a result, dipole moments obtained from composite methods do not reflect the same level of theoretical accuracy as their corresponding energy values. Following *Head-Gordon and Hait (2018)*, the errors were regularized using eq 10.^42^ Where μ_*i*_ is the dipole moment of the molecule, μ_ref_ is the reference dipole moment, and the denominator is either the μ_ref_ or 1, whatever is larger. This was used to prevent a single species from dominating or skewing analysis. Specifically, molecules with small dipole moments contribute absolute errors, while those with large dipole moments are evaluated using relative errors, allowing for more balanced comparison across the data set. The regularized errors were used to find MUE, RMSD, and max absolute error.

10 $$\mathrm{Regularized}\;\mathrm{error}=\frac{\mu_{i}-\mu_{\mathrm{ref}}}{\max\left(\mu_{\mathrm{ref}},1D\right)}\times 100\%$$

## 3 Results and Discussion

### 3.1 Benchmarking Calculations: Thermochemical Properties

#### 3.1.1 Basis Set Evaluation: BDEs

We assessed basis-set effects on BDEs using PFBA and PFBS in both protonated and deprotonated forms using def2-QZVPD as the reference (Figures 1 and S1). Across the Karlsruhe def2 series, the basis set alone can shift BDEs by more than 12 kcal/mol, underscoring that basis-set choice is a first-order source of uncertainty. Diffuse functions (denoted by ‘‘D”) are essential, especially for deprotonated anions. Without them, even a large quadruple-ζ basis set (def2-QZVP) overestimates the strength of the bond between the acidic headgroup (COOH/COO^–^ in PFBA or SO_3_H/SO_3_^–^ in PFBS) and the α-carbon (C­(α)) by >2 kcal/mol. In protonated forms, diffuse functions have a clear but smaller benefit. For example, switching from def2-TZVP to def2-TZVPD reduces the maximum error from −1.34 kcal/mol to −0.61 kcal/mol for PFBS, and from −1.31 kcal/mol to −0.52 kcal/mol in PFBA. An additional polarization function (PP) brings little additional improvement once diffuse functions are present. While relative performance depends on implementation, hardware, and basis set functionals, our comparisons (Table S1) indicate that adding diffuse functions at the triple-ζ level delivers near-quadruple-ζ accuracy at substantially lower cost. On the basis of balancing accuracy and cost, def2-TZVPD emerges as a practical default for PFAS enthalpy properties.

**Figure 1:**
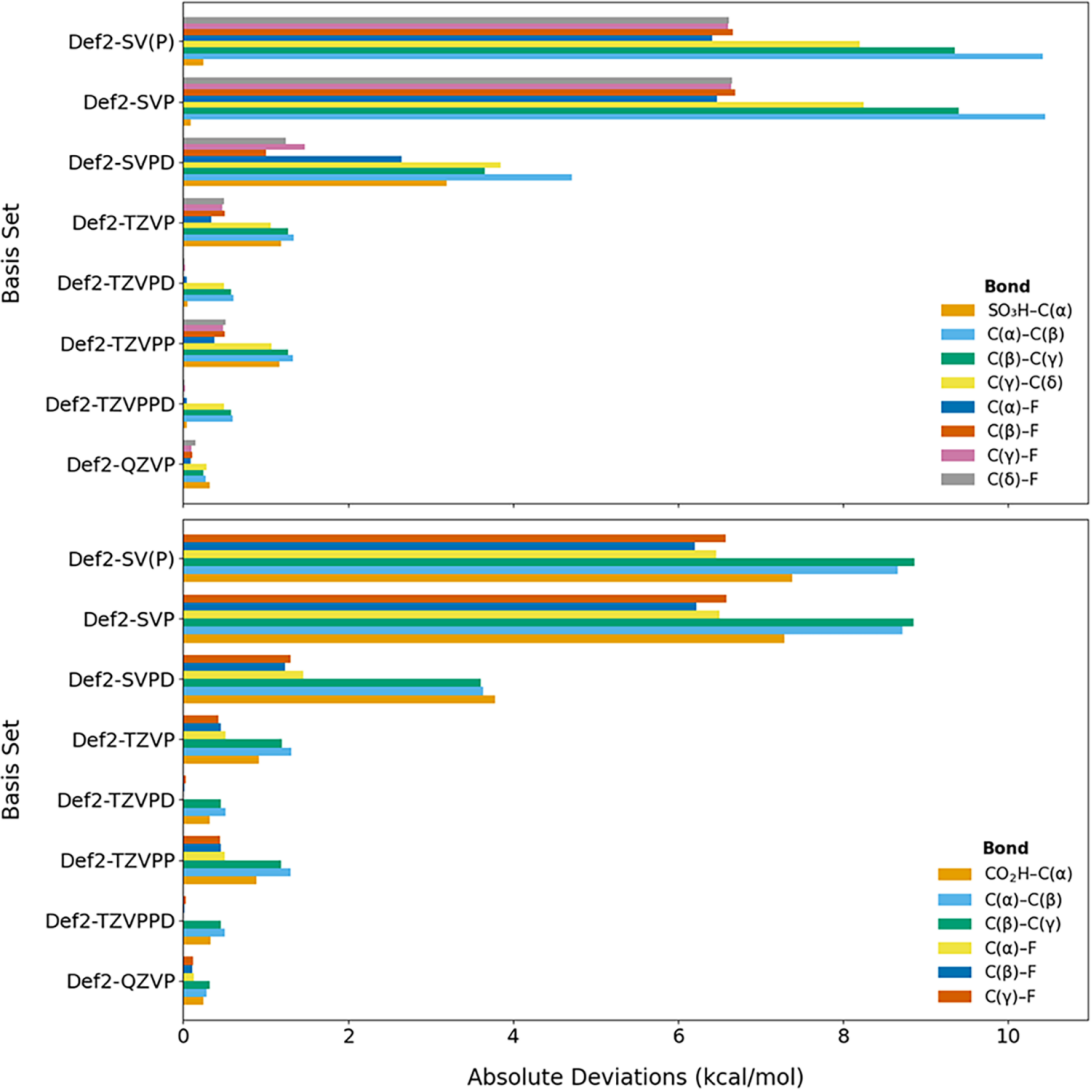
Basis set dependence of BDEs for PFBS (top) and PFBA (bottom) in protonated forms using the Karlsruhe def2 series as compared to def2-QZVPD. The def2-TZVPD basis set was selected as the optimal level of theory, balancing efficiency and accuracy.

#### 3.1.2 Method Evaluation via Experimental BDE Data Set (Data Set 1)

Experimental BDEs are available for a small set of C–H/C-F bonds (CH_4_, CH_3_F, CH_2_F_2_, CHF_3_, and CF_4_). On this set (*n* = 8), G3, G4, and W1BD are statistically indistinguishable, with MUE of 0.39, 0.69, and 0.55 kcal/mol, respectively (Figure S2), within typical experimental uncertainty (±1 kcal/mol). Thus, experiment alone does not separate the composite references. Prior benchmarking of enthalpies of formation by Simmie and Somers found G3 ≈ G4 > W1BD > CBS-APNO > CBS-QB3 for C*_x_* H*_y_* O*_z_* molecules.^29^ Similarly, G4 and CBS-QB3 compared against 29 fluorinated and nonfluorinated species yielded mean absolute deviations of 0.6 and 2.7 kcal/mol, respectively.^32^ Specifically, BDE benchmarking in halogenated species showed that G4 outperformed G3, G3­(MP2), and CBS-QB3, although ωB97X-D/6–311++G­(d,p) was identified as the top performer in that particular data set.^99^ In our experimental data set ωB97X-D3 underperformed (2.13 kcal/mol MUE) and was not considered for reference methods. Due to the limited available BDE data for PFAS-relevant systems, broader thermochemical benchmarking evidence was considered for selection of a reference method for PFAS benchmarking. While enthalpies of formation were not explicitly computed within the scope of this work, the provided database enables calculation of enthalpies of formation as well as other thermodynamic properties. The accurate determination of enthalpy of formation requires careful treatment of reference schemes and protonation states; therefore, this study focuses on bond dissociation energies as a more direct and consistently comparable metric, with enthalpy of formation benchmarking representing valuable future work. Given its favorable balance between accuracy and computational cost relative to W1BD, G4 was selected as the reference for PFAS benchmarking (Data Set 2). Limited checks with CCSD­(T)/aug-cc-pVTZ//CCSD/aug-cc-pVTZ (*n* = 4) gave MUE/RMSD 1.49/1.54 kcal/mol, consistent with the composites (Figure S1 and Table S2). To extend high-accuracy coverage beyond canonical CCSD­(T), DLPNO–CCSD­(T) and DLPNO–CCSD­(T)-F12 single point energies paired with B1 (MP2/aug-cc-pVTZ) or G4 thermochemistry also achieved within ± 1 kcal/mol MUE on this set.

#### 3.1.3 Method Evaluation for PFAS BDEs Relative to Theory (Data Set 2)

On the expanded PFAS/PFAS-like set, G3 and G4 no longer agreed. Relative to G4, G3 showed MUE of 3.82 kcal/mol, RMSD 5.07 kcal/mol, and a maximum absolute deviation of 19.14 kcal/mol, with systematically higher BDEs (Figure 2 and Table S3). The largest discrepancy occurred for O–H cleavage in triflic acid (SO_3_HCF_3_). Although these methods typically agreed for nonfluorinated systems, the PFAS environment amplified their differences, making the choice of reference nontrivial. For this work, G4 was retained as the primary reference method for evaluating lower-cost approaches due to its previous uses as a reference, as described in Section 3.1.2. Range-separated functionals with advanced correlation treatment ωB97X-V and ωB97X-2 were top performers, with MUE values of 1.84 and 1.87 kcal/mol and RMSD values of 3.77 and 2.22 kcal/mol, respectively. MN12SX also ranked among top performers with 1.73 MUE and 2.19 kcal/mol RMSD. M06 and the meta-GGA B97M-V were close behind (MUE 2.04 and 3.12 kcal/mol; RMSD 2.47 and 5.74 kcal/mol), offering attractive cost-accuracy options for larger data sets. Notably, the functionals based on Becke’s 1997 power-series framework with modern optimization and advanced correlation treatment, including ωB97X-V, ωB97X-2, ωB97X-D3 (MUE 2.72 kcal/mol; RMSD 4.70 kcal/mol), ωB97M-V (MUE 2.81 kcal/mol; RMSD 6.82 kcal/mol), and B97M-V, all ranked among top performers within their respective DFT rungs. The older B97-D functional, which uses D2 dispersion performed poorly (MUE 8.03 kcal/mol), substantially worse than the best GGA (BP86, MUE 6.04 kcal/mol).

**Figure 2:**
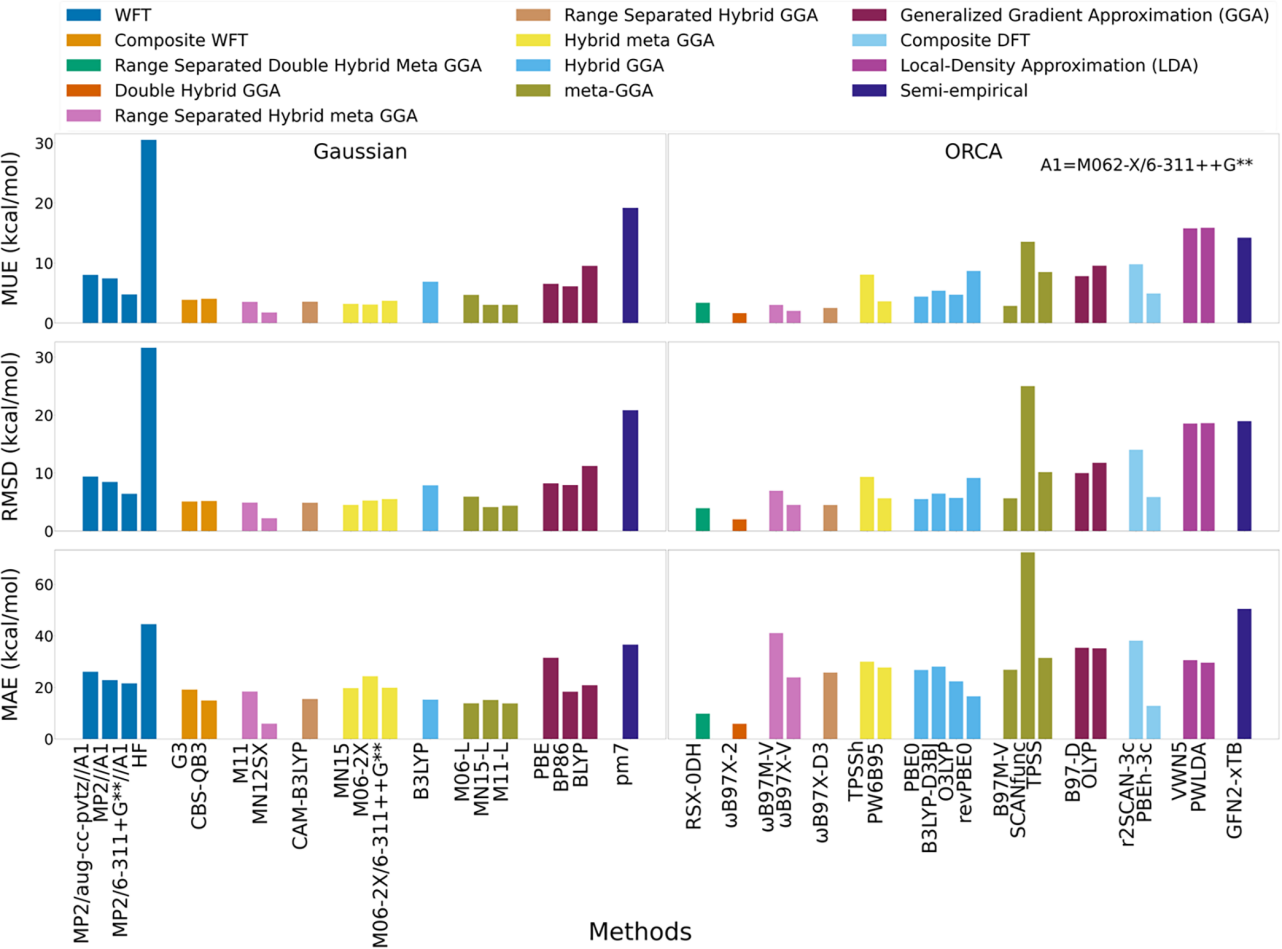
MUE, RMSD, and Maximum Absolute Error (MAE) for predicted BDEs across neutral PFAS and PFAS-like molecules relative to G4 (*n* = 87).

In contrast, MP2/aug-cc-pVTZ, evaluated on a reduced subset (*n* = 45) (see Figure S3 and Table S3) due to computational cost, overestimated BDEs vs G4 (MUE 5.52; RMSD 6.45 kcal/mol). Single-point MP2 energies with M06–2*X*/6–311++G** thermal corrections and various basis sets (aug-cc-pVTZ, def2-TZVPD, 6–311+G**) allowed simulation of larger PFAS but gave worse agreement with G4, indicating MP2 was not competitive for PFAS BDEs at comparable cost. DLPNO–CCSD­(T) and its variants, tested on the same subset, improved performance substantially. DLPNO–CCSD­(T)-F12 with B1 thermochemistry achieved MUE/RMSD 1.43–1.74/1.81–2.37 kcal/mol using cc-pVTZ and aug-cc-pVQZ bases, reflecting faster basis-set convergence and accuracy from the F12 treatment. This suggests strong potential for DLPNO–CCSD­(T)-F12 in PFAS BDE calculations. Semiempirical methods performed poorly relative to G4 and are not recommended. PM7 underestimated BDEs (MUE = 19.0; RMSD = 20.6 kcal/mol), while GFN2-xTB overestimated them (MUE = 14.5; RMSD = 19.4 kcal/mol) (Figure S3 and Table S3).

For deprotonated (anionic) PFAS (*n* = 43), rankings remained qualitatively similar but absolute errors increased (Figure S4 and Table S4). This partly reflects the smaller data set (*n* = 43 vs 87) and the structural instabilities of gas-phase anions. Discrepancies between G4 and both G3 and MP2 were amplified by the SO_3_^–•^ fragment. G3 and MP2 predict a planar SO_3_^–•^ geometry, artificially increasing BDEs for all associated bonds. This highlights the need for caution in optimizing anionic PFAS, as subtle geometric differences can systematically bias energies. While DLPNO–CCSD­(T)-F12 improved neutral PFAS predictions, this advantage did not extend to anions, likely due to geometry-driven MP2 errors. Evaluating DLPNO–CCSD­(T)-F12 with alternative thermochemical corrections for anionic PFAS is warranted but beyond the scope of this work.

### 3.2 Benchmarking Calculations: Electronic Properties

Electronic properties of PFAS influence numerous physicochemical behaviors relevant to their environmental and biological fate. Recent work has used PFAS dipole moments to develop quantitative structure–property relationship (QSPR) models predicting organic-carbon–normalized sorption coefficients at the B3LYP/6–31G* level.^100^ Electron affinities of radical PFAS represent key descriptors for diradical intermediates involved in degradation. Vyas and Hoomissen (2019) showed that the anionic diradical PFBA^–^ species is thermodynamically more favorable to react with water than the single-radical pathway.^22^ Expanding accurate data sets of electrostatic descriptors will improve future predictions of PFAS fate, transport, bioaccumulation, and toxicity, and clarify degradation mechanisms.

#### 3.2.1 Basis Set Evaluation: Dipole Moment

Dipole moments were benchmarked with the M06–2X functional using both the Pople and Karlsruhe (def2) basis-set families. M06–2X was selected for this basis set comparison based on method screening with def2-TZVPD, which identified M06–2X as the top performer (Section 3.2.2). Because computational cost scales with basis-function count, average basis-function numbers were evaluated and referenced to def2-QZVPD (Figures 3, S5 and Tables S5, S6). In the Pople family, a second ‘‘+/*” (diffuse/polarization) corresponds to only hydrogen atoms, so it had little effect on this PFAS set (few hydrogen atoms). Accuracy improved notably with additional d- and f-functions (6–311++G­(2df,2pd)), approaching def2-TZVP but still lagging behind the accuracy of def2-TZVPD. Within the def2 family, accuracy increased systematically with basis-set size, though extra polarization functions offered minimal further benefit. These results align with prior broad-set studies showing def2 bases outperform Pople double- and triple-ζ sets for dipoles.^42,101^ In our PFAS specific evaluation, def2-TZVPD was preferred for dipole moments. If Pople sets are used, we recommend restriction to at least triple-ζ with diffuse and polarization, noting that 6–311++G** also underperformed for BDEs when compared to def2-TZVPD with M06–2X (see Section 3.1.3).

**Figure 3:**
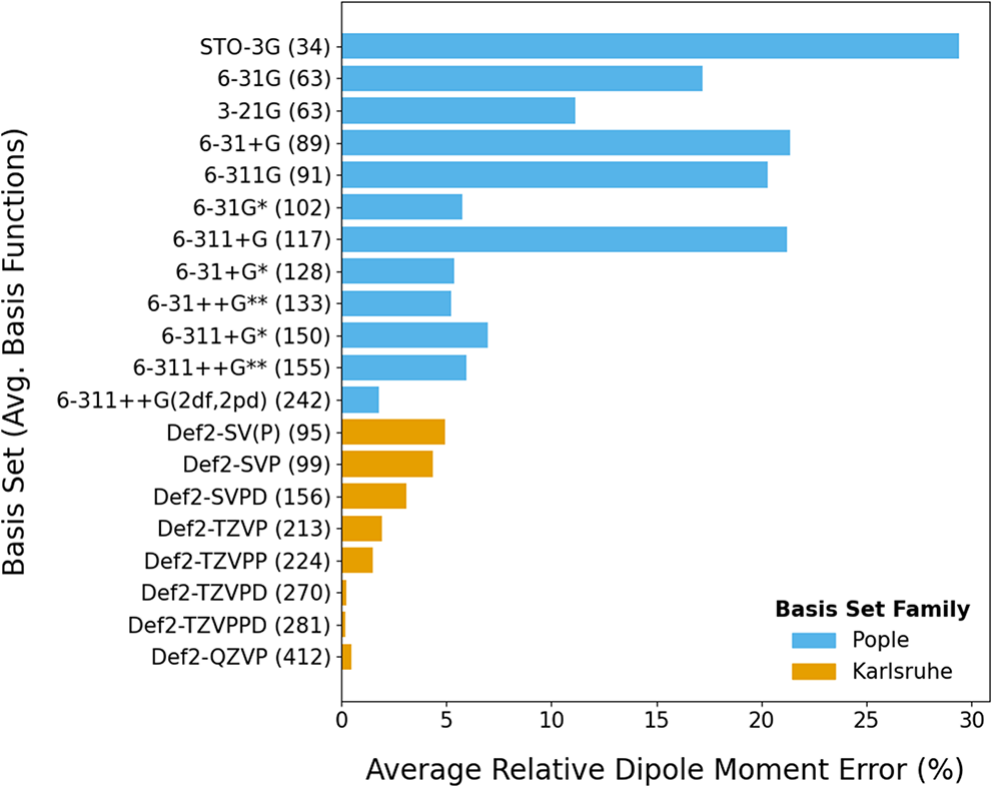
Average dipole moment error relative to def2-QZVPD for neutral PFAS and PFAS-like molecules using M06–2X (*n* = 94). The basis set chosen from this evaluation was def2-TZVPD.

#### 3.2.2 Method Evaluation to Experimental Dipole Moment (Data Set 1)

In order to select an appropriate benchmark for PFAS, a small subset of molecules that would resemble PFAS structures (fluorination, head groups, etc.) was constructed by combining available experimental values (*n* = 24) with high-quality CCSD­(T)/CBS reference values of fluorinated species from Hait and Head-Gordon (2018) (*n* = 7). Figure S6 and Table S8 summarize the relative MUE, relative RMSD, and relative maximum absolute error (MAE) for all methods tested. Prior work by Hait and Head-Gordon noted that MP2 performs poorly with spin-polarized molecules (RMSE 54.63%),^42^ unless the RMP2 is used (RMSE 9.84%). Additionally, MP2/aug-cc-pvtz was specifically reported to have a regularized RMSE of 0.170.^101^ Although MP2 is known to struggle with breaking spin symmetry, only 6 radical molecules were included in this evaluation data set, and spin contamination (via S2) was explicitly checked before the molecule was included, which may explain the relatively good performance of MP2 at 2.95% MUE and 4.82% RMSD. The semiempirical PM7 method performed worst, despite a modest (∼10%) improvement relative to broader data sets.^102^ In this data set, M06–2X performed best, outperforming CCSD/aug-cc-pvtz with relative MUE, RMSD, and max absolute error percentages of 2.07%, 3.40%, and 10.78%, respectively. In comparison, M06–2X had an RMSE of 7.94% with the Hait and Head-Gordon full 200-molecule data set. Based on this performance, M06–2X was selected as the reference method for evaluating the larger PFAS data set (Data Set 2).

#### 3.2.3 Method Evaluation Relative to Theory: Dipole Moment (Data Set 2)

The data set comprised 62 radical and 15 closed-shell PFAS or PFAS-like molecules, spanning fluorotelomers, PFCAs, PFSAs, and fluorinated alkyl chains. The deprotonated set added 51 species (35 open-shell, 16 closed-shell) from the same classes, excluding alkyl chains. Open-shell systems are sensitive to electron delocalization, which challenges correlated methods such as MP2.^42^ This chemically diverse PFAS set enables transferable benchmarking across PFAS families and meaningful comparisons to the M06–2X/def2-TZVPD baseline, rather than establishing absolute accuracy within the PFAS data set. Together with the independent validation presented in Data Set 1 (Section 3.2.2), the relative performance trends observed here provide complementary guidance for selecting appropriate dipole-moment methods for PFAS applications, depending on the intended use.

Figures 4 and S7–S11 (Tables S8–S9) report relative MUE, RMSD, and MAE with respect to the M06–2X/def2-TZVPD baseline, allowing assessment of functional consistency across PFAS chemical space. For most functionals, spin-polarized and nonspin-polarized subsets showed similar MUE and RMSD, though some methods (e.g., revPBE0) yielded large, method-specific deviations. MN15 most closely tracked M06–2X (3.40% relative MUE, 6.06% relative RMSE) and performed comparably on the experimental set (3.34% relative MUE); in more wide-ranging chemical space MN15 was reported to have a 10.53% RMSE (Hait–Head-Gordon data set).

**Figure 4:**
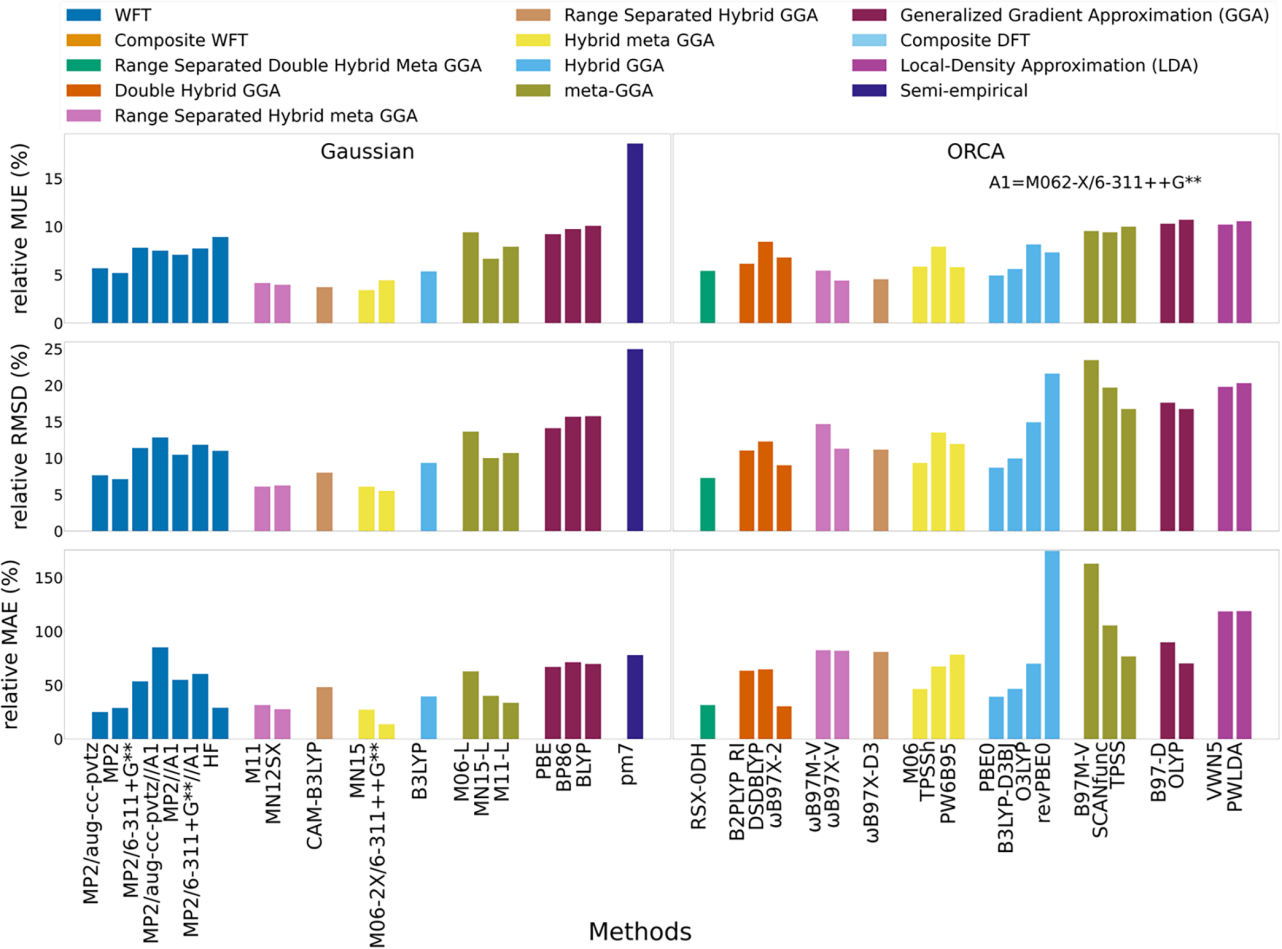
MUE, RMSD, and MAE in dipole moments predicted across neutral PFAS and PFAS-like molecules relative to M06–2X/def2-TZVPD (*n* = 77).

Trends aligned more strongly with individual functionals than with entire Jacob’s ladder tiers. However, under- or overestimation seemed to be more ladder-tier based (Figure S9). The amount of HF exchange content was a clear driver. DFT hybrids generally outperformed their parent semilocals: M06, M11, MN15 improved relative to their -L counterparts (0% HF); PBE0 (25% HF) outperforms PBE (0% HF); and TPSSh (10% HF) modestly improved on TPSS. An exception was revPBE0, which diverged from PBE0, underscoring that functional details matter in addition to HF fraction. Given the prevalence of delocalized radicals in PFAS chemistry, adequate (long-range) HF exchange likely aids accurate dipole prediction.

Rankings for deprotonated anions were broadly consistent with those of the neutral systems, though errors increased, with radical anions showing the largest deviations (Figures S10–S11). A notable artifact was MP2’s prediction of symmetric SO_3_^–^ geometry (zero dipole), which inflated errors; robust diffuse functions and careful geometry protocols are essential. MN15 remained among the top performers (MUE = 3.24%, RMSD = 5.43%), slightly surpassed by CAM-B3LYP (MUE = 2.47%, RMSD = 6.88%). Both are hybrids, but CAM-B3LYP (hybrid GGA) is lower on the ladder than MN15 (hybrid meta-GGA), though the exact difference depends on implementation. Since acid PFAS readily deprotonate, selecting a method consistent across neutral and anionic forms is particularly valuable.

#### 3.2.4 Method Evaluation to Experimental: Ionization Potential and Electron Affinity (Data Set 1)

Both vertical and adiabatic ionization potentials (IP) and electron affinities (EA) were benchmarked; only adiabatic results are discussed here, while vertical values are available in the database. Thirty C/F/O/H/S containing molecules were evaluated to identify a suitable method for PFAS. For ionization potential, G4 yielded the lowest errors (MUE = 0.18 eV; RMSD = 0.47 eV; Figure S12 and Table S10) and remained computationally scalable for short-chain PFAS. In contrast, CCSD­(T)/aug-cc-pVTZ//CCSD/aug-cc-pVTZ and DLPNO–CCSD­(T)-based methods showed reliability issues due to spin contamination and elevated *T*_1_ diagnostics for several species (CO, CO_2_, SO_3_, HO_2_^•^, OH^•^). These were excluded, reducing their suitability as general references. The OH^•^ was a recurrent outlier across all methods. Within DLPNO–CCSD­(T)-F12, increasing the basis from cc-pVTZ to cc-pVQZ had negligible impact (MUE 0.22 to 0.20 eV; RMSD 0.46 to 0.48 eV). On a smaller 16-molecule test set for electron affinity, G4 again performed well (MUE = 0.13 eV; RMSD = 0.28 eV; Figure S13 and Table S11). High-level WFT methods continued to show problematic *T*_1_ values for anionic species (F^–^, H^–^, HO_2_^•^, OH^•^), while SO_3_H exhibited >1 eV errors for many methods. A limited anion-detachment subset (CO_2_CFH_2_, CO_2_CHF_2_, PFBA^–^; Table S12) was also tested. WFT convergence was difficult; several DFTs matched experiment closely (ωB97X-2 and RSX-0DH: MUE 0.04/0.07 eV; RMSD 0.05/0.08 eV), but the sample is too small to be definitive. Accordingly, G4 was adopted as the reference method for Data Set 2 for IP and EA.

#### 3.2.5 Method Evaluation to Theory: Ionization Potential (Data Set 2)

Adiabatic IPs were computed for 62 protonated and protonated-radical PFAS/PFAS-like molecules using G4 as the benchmark (Figures 5, S14 and Table S13). Among composites, CBS-QB3 tracked G4 best (MUE 0.11; RMSD 0.14 eV), outperforming G3 (0.25; 0.42 eV) and consistent with BDE trends. MP2 and lower-rung GGA/LDA/meta-GGA methods exceeded 0.3 eV MUE, while semiempirical methods performed worst, particularly GFN2-xTB without IPEA-xTB reparameterization^103^ (MUE 4.6 eV; RMSE 4.7 eV). DLPNO–CCSD­(T)-F12 markedly improved over its MP2-based thermochemistry (MUE 0.15–0.16; RMSD 0.34–0.35 eV), with minimal basis-set dependence among def2-TZVPD, cc-pVTZ, cc-pVQZ, and aug-cc-pVQZ. Among DFTs, M06–2X and CAM-B3LYP performed comparably well (MUE 0.13–0.15; RMSD 0.26 eV). Using 6–311++G** slightly degraded M06–2X accuracy (MUE 0.21; RMSE 0.33 eV) relative to def2-TZVPD. Outliers clustered in chain-terminal radicals (e.g., ^•^CH_2_CF_2_CF_2_CF_2_CF_3_, SO_3_HCF_2_CF_2_^•^, and SO_3_HCF_2_CF_2_CF_2_^•^), pushing MAE greater than 1 eV across methods. Outside of radical PFAS, MUE and RMSD values dropped (Figure S15). Radicals accounted for the greatest increase in MAE deviations, but only raised MUE and RMSD slightly.

**Figure 5:**
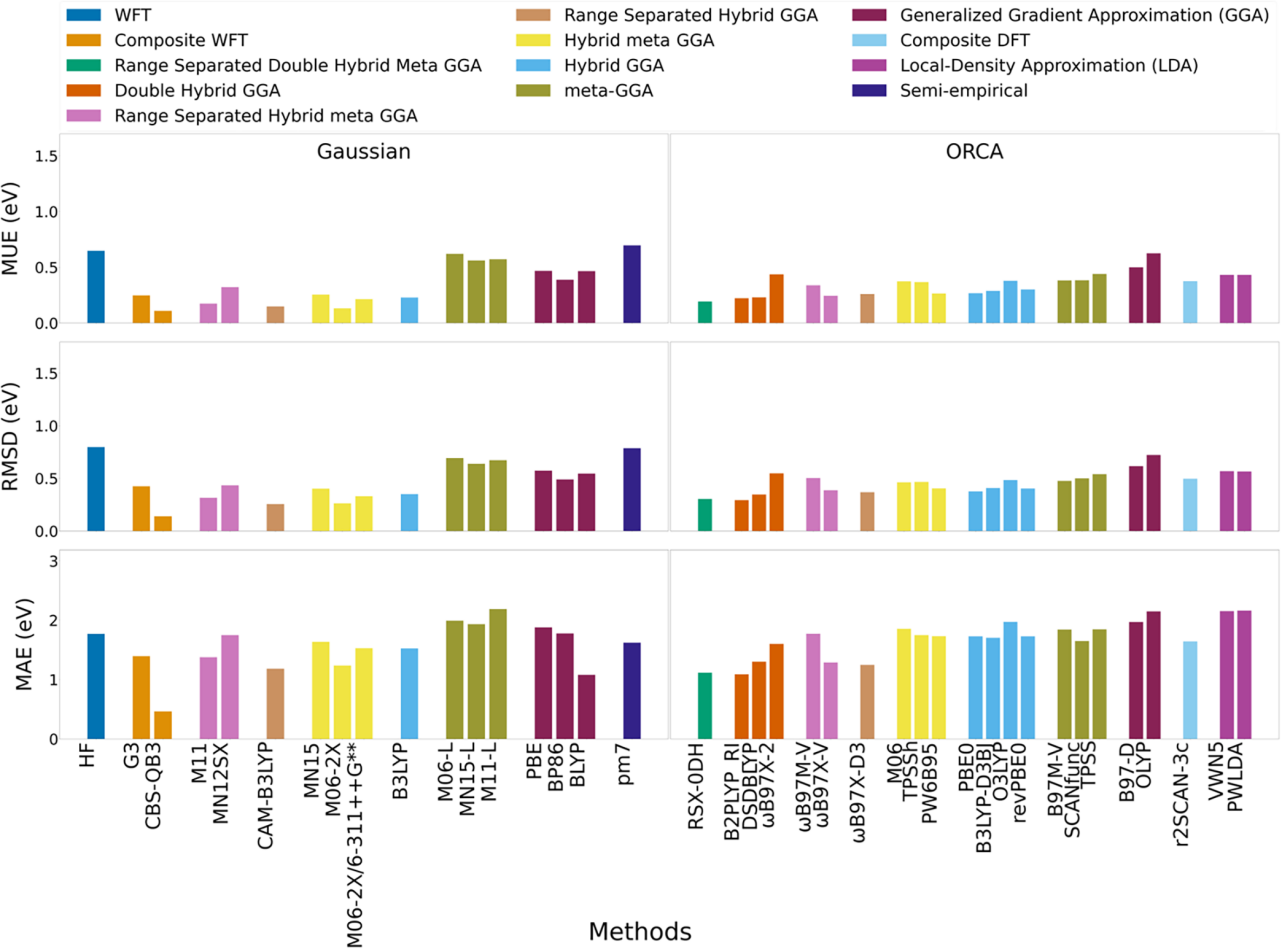
MUE, RMSD, and MAE in ionization potential predicted across neutral and neutral radical PFAS and PFAS-like molecules relative to G4 (*n* = 62).

For 39 deprotonated and radical-deprotonated cases, several methods showed imaginary frequencies in the gas phase; affected molecules were excluded before statistical analysis (Figure S16 and Table S14). Rankings generally mirrored neutrals, with slightly higher errors (e.g., M06–2X MUE 0.13 to 0.17 eV). The modern B97 framework functionals improved relative to neutrals, with the exception of ωB97X-D3 (MUE neutral: 0.26 eV; MUE anionic: 0.394 eV). CAM-B3LYP, M06–2X, and DLPNO–CCSD­(T)-F12 all remained top performers. Compared to the small experimental subset, RSX-0DH and ωB97X-2 no longer matched G4 as closely (MUE/RMSD = 0.26/0.34 eV and 0.28/0.60 eV, respectively). While experimental data are limited for anionic PFAS, the best practice is to combine overall performance across all data sets. Evaluating methods based on consistency across both neutral and anionic systems, top performers were M06–2X, ωB97M-V, and CAM-B3LYP.

#### 3.2.6 Method Evaluation to Theory: Adiabatic Electron Affinity (Data Set 2)

Seventy-six protonated and protonated-radical PFAS/PFAS-like molecules were evaluated against G4 (Figure 6, Table S15, and Figure S17). Composite methods were similar to one another (CBS-QB3 G3; MUE 0.20/0.23 eV; RMSE 0.47/0.49 eV). No DFT achieved RMSD < 0.24 eV, indicating a need for improvement. MUEs spanned 0.24–0.42 eV. CAM-B3LYP and MN15 were the most consistent DFTs (both MUE 0.24 eV; RMSE 0.49/0.48 eV). Separating radicals from nonradicals showed slightly better aggregate performance for radicals (Figure S18). However, most of the largest errors stemmed from the SO_3_HCH_2_CF_2_^•^ radical, with deviations well exceeding 2 eV.

**Figure 6:**
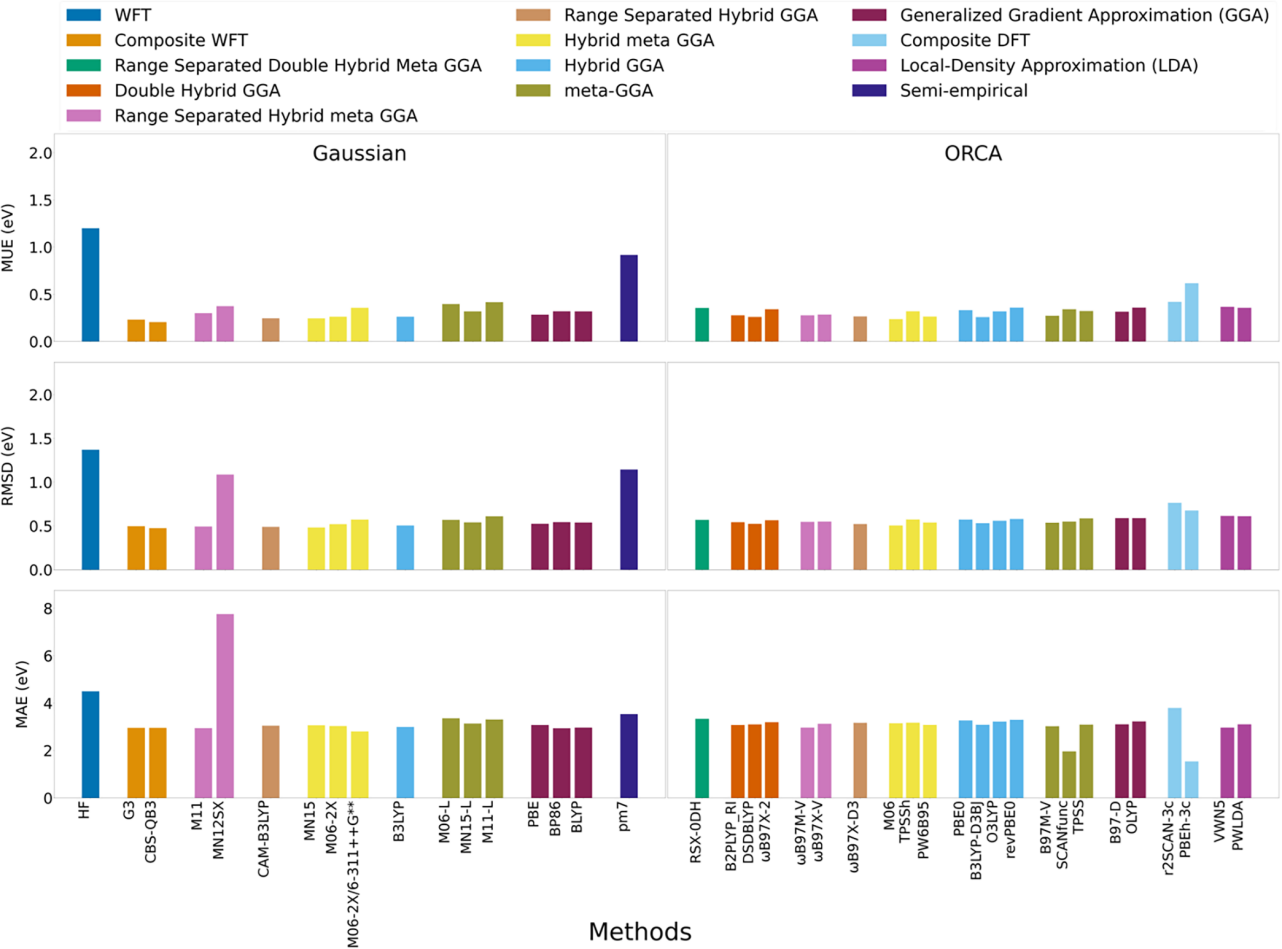
MUE, RMSD, and MAE in electron affinity predicted across neutral and neutral radical PFAS and PFAS-like molecules relative to G4 (*n* = 76).

For 40 deprotonated and deprotonated radical cases (Figure S19 and Table S16), convergence issues and imaginary frequencies were frequent in the anionic gas phase. Relative to neutrals, errors increased. CAM-B3LYP (MUE 0.36; RMSE 0.93 eV) and MN15 remained relatively top performers (0.23; 0.38 eV), while MN12-SX yielded a low MUE (0.14 eV) but suffered from incomplete coverage (31/40 completed). Overall, electron affinity was more difficult to model for PFAS in both neutral and anionic forms than other properties.

### 3.3 Further Considerations: Radical Delocalization

Radical PFAS play a central role in degradation mechanisms and environmental persistence. The way a method models the unpaired electron directly impacts the accuracy of predicted reactivity, making it important to understand how different computational approaches characterize radical delocalization. Population analysis methods give insight into the degree of delocalization across a molecule through orbital-based, volume-based, and potential-derived charges approaches.^104^ In this work we focus on orbital-based approaches which are widely implemented, low cost, and useful for qualitative insights.^105^ Among those, Mulliken^106^ and Löwdin^107^ analyses are broadly implemented and computationally efficient, but both methods are known to be strongly basis set dependent,^104^ while NBO^108^ offers more chemically accurate orbitals at a slightly higher cost. Figure 7 (Table S17) compares Mulliken and Löwdin population analyses to NBO for PFAS and PFAS-like molecules using M06–2X/def2-TZVPD. While Löwdin had a tendency to underestimate the spin density of the radical atom, Mulliken only slightly overestimated them, particularly around 0.85, compared to NBO. For this small PFAS radical set, Mulliken spin populations showed a stronger correlation with NBO results (*R* ^2^ = 0.73) than Löwdin populations did (*R* ^2^ = 0.60). Although all three methods captured different trends using M06–2X/def2-TZVPD, NBO was selected as the basis for the qualitative analysis of delocalization in both protonated and deprotonated PFAS radicals.

**Figure 7:**
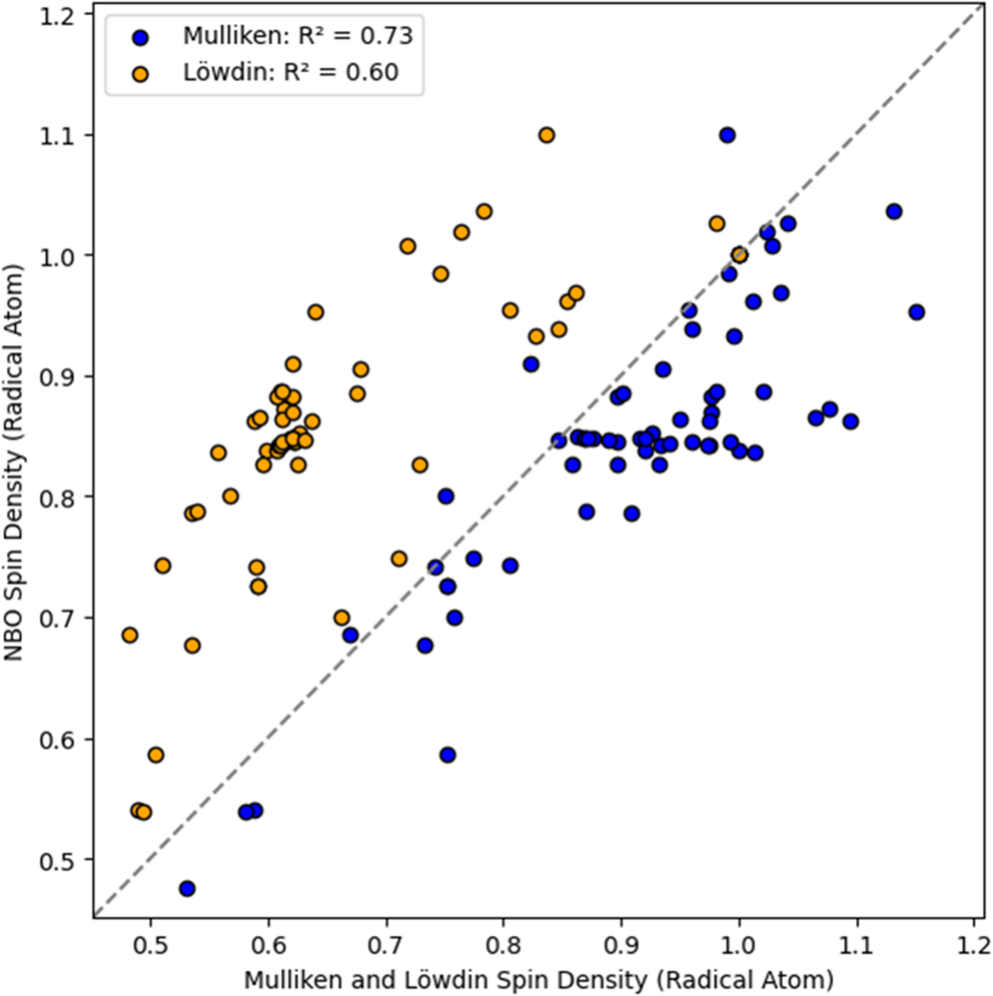
NBO versus Mulliken and Löwdin Spin density population analyses of radical atom on neutral PFAS and PFAS-like compounds using M06–2X/Def2-TZVPD (*n* = 65).

Figures 8 and 9 give insight into how an unpaired electron distributes across PFAS radicals derived from PFBA and PFBS by specific bond cleavage. For each radical, the spin population on the radical center and along the backbone reveals clear shifts between more localized and more delocalized cases. While many additional PFAS radicals exhibit delocalization (PFAS_Database, SITables S18 and S19), PFBA and PFBS in both their protonated and deprotonated forms were chosen to highlight how electron spin density changes with bond type, protonation state, and distance from the radical atom.

**Figure 8:**
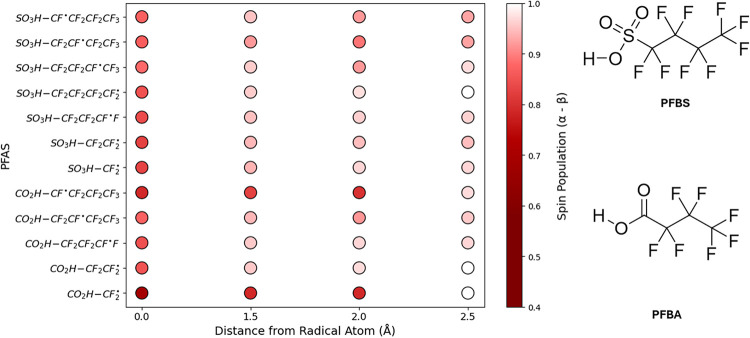
NBO Spin population analysis of defluorinated and C–C cleaved bonds of PFBS and PFBA by distance from the radical atom.

**Figure 9:**
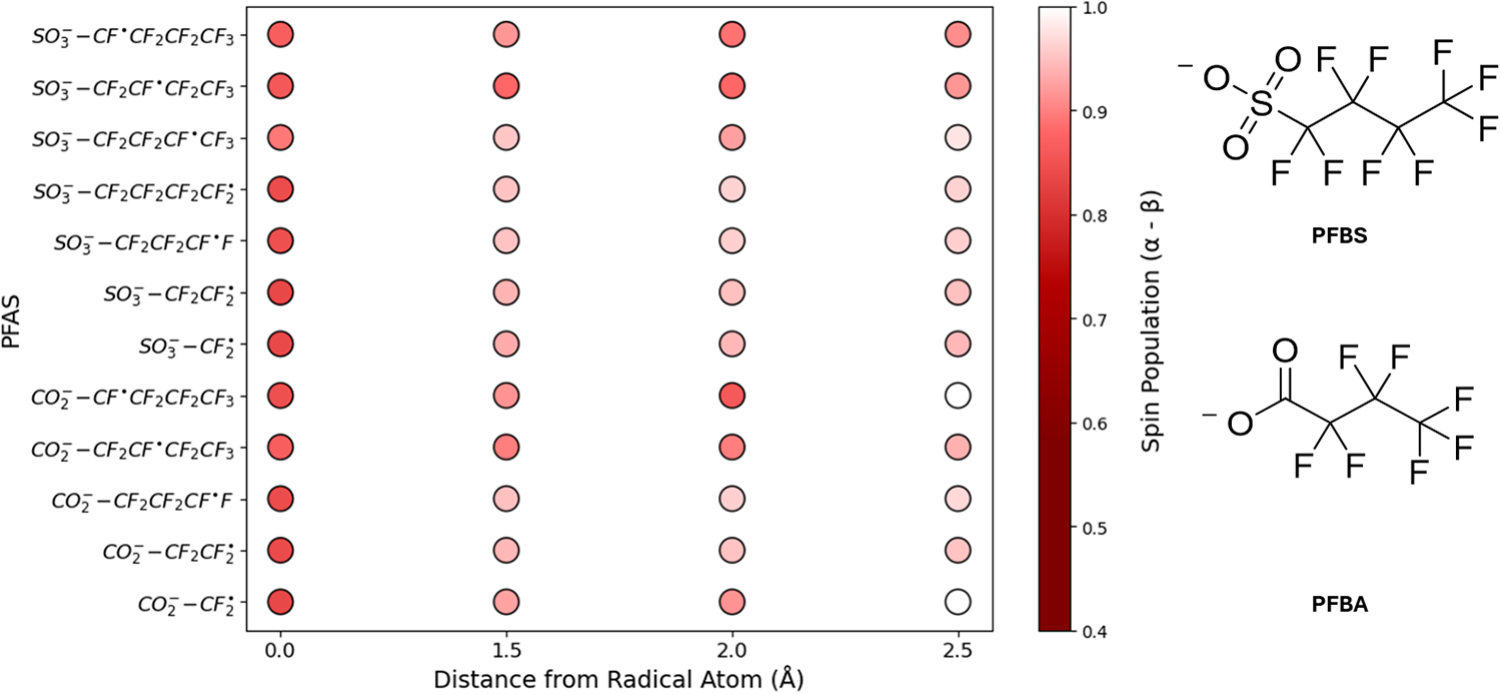
NBO Spin population analysis of defluorinated and C–C cleaved bonds of PFBS^–^ and PFBA^–^ by distance from the radical atom.

Higher local spin populations generally indicate a site of increased radical reactivity, while greater delocalization can reduce site specific reactivity. NBO analysis showed that all radicals examined displayed at least some degree of delocalization along the perfluorinated backbone, regardless of protonation state. Among the PFBA and PFBS fragments, the CO_2_H–CF_2_^•^ radical exhibited the largest extent of delocalization. Protonated PFAS, in general, seemed slightly more susceptible to larger degrees of delocalization than their deprotonated counterparts. Overall, these observations illustrate how modest variations in molecular structure can alter the distribution of unpaired electron density, emphasizing the need to consider delocalization when characterizing PFAS radical behavior.

## 4 Conclusion

This benchmarking study established G4 as a practical reference method for predicting PFAS properties, while recognizing that limited experimental data leave room for continued refinement; the database provided as a key contribution of this work is meant to enable such ongoing refinement. Key findings and recommendations are summarized below:

**Reference level:**

- G4 provides a practical benchmark for PFAS properties.
- G3 diverges significantly for BDEs, unlike prior enthalpy of formation studies, highlighting the need for more experimental data.

**Geometry optimizations:**

- Small structural differences between methods can strongly impact predicted properties, stressing the need for careful optimization.

**Basis set recommendations:**

- *def2-TZVPD* offers a reliable balance of cost and accuracy for both protonated and deprotonated PFAS.
- Depending on property and tolerance, smaller and faster basis sets may suffice, but the use of diffuse functions is advisable.
- Use caution with Pople basis sets for electronic properties, as errors increase relative to Karlsruhe/Dunning families.

**High-level methods:**

- DLPNO–CCSD­(T)-F12 is promising for large PFAS but should be paired with a thermal correction protocol other than MP2/aug-cc-pVTZ.
- MP2 is not recommended for PFAS modeling due to large inaccuracies relative to its computational cost.

**Density functionals:**

- Overall, ωB97X-V is a reasonable choice when calculating multiple properties with a single method. It excels in thermodynamic properties, and remains within the moderate-to-strong range in electronic properties across protonated and deprotonated PFAS.
- **Homolytic BDEs**: ωB97X-2 and MN12-SX are among the most accurate for BDEs. M06 or B97M-V are both cost-efficient, accurate choices for both protonated and deprotonated PFAS.
- **Dipole moment/ionization potential:** M06–2X and CAM-B3LYP perform exceptionally well and are recommended for specific IP or dipole moment studies; RSX-0DH and ωB97X-2 achieved < 0.1 eV MUE for IP with three deprotonated experimental PFAS and PFAs-like molecules. However, this did not translate to the G4 benchmarking set.
- **Electron affinity:** No DFT achieved < 0.24 eV MUE for protonated species; higher-level methods and additional basis-set exploration are recommended. For deprotonated species (diradicals), MN12SX performed closest to G4.

**Deprotonated PFAS:**

- Gas-phase modeling remains challenging due to charge delocalization and shallow vibrational modes.
- Continuum solvation can stabilize such systems and improve convergence, though it was excluded here for benchmarking consistency.

Together, these benchmarks and the accompanying database, which is intended as living document, provide a practical roadmap for more reliable PFAS calculations, a high-quality training resource for next-generation predictive models, and an evolving foundation for refining best practices as new experimental data emerge. Areas of particular interest to the community include rigorous benchmarking of enthalpies of formation and systematic evaluation of protonation and conformational effects, which could be enabled by the breadth of thermochemical and electronic data provided across methods to improve predictive modeling of PFAS behavior.

## Supplementary Material





## Data Availability

All data used in this study are available in a publicly accessible GitHub repository at https://github.com/Ng-Lab-Group/PFAS_Database. The repository contains machine-readable CSV files that include molecular identifiers, SMILES strings, Cartesian geometries, and calculated thermochemical and electronic properties used in this work.

## Notes

SI 1: Thermodynamic properties including Basis Set Comparison (Figure S1, Table S1), Experimental BDE Comparison (Figure S2), BDE Performance Relative to Theory (Figures S3, S4). Electronic Properties, including Dipole Moment Basis Set Analysis (Figure S5), Experimental Dipole Moment Comparison (Figure S6), Dipole Moment Performance Relative to Theory (Figures S7–S11), Electron Affinity and Ionization Potential Experimental Comparison (Figures S12, S13), Ionization Potential Performance Relative to Theory (Figures S14–S16) and Electron Affinity Performance Relative to Theory (Figures S17–S19) (PDF)
SI 2: Excel file with all supporting statistical tables (Tables S2–S19 and experimental data on neutral compounds) (XLSB)

## References

[1] Buck R. C., Korzeniowski S. H., Laganis E., Adamsky F. (2021). Identification and
classification of commercially relevant per- and poly-fluoroalkyl
substances (PFAS). Integr. Environ. Assess.
Manage..

[2] Glüge J., Scheringer M., Cousins I. T., DeWitt J. C., Goldenman G., Herzke D., Lohmann R., Ng C. A., Trier X., Wang Z. (2020). An overview of the uses of per- and polyfluoroalkyl substances (PFAS). Environ. Sci.: Processes Impacts.

[3] Cousins I. T., Kong D., Vestergren R. (2011). Reconciling
measurement and modelling
studies of the sources and fate of perfluorinated carboxylates. Environ. Chem..

[4] Armitage J. M., MacLeod M., Cousins I. T. (2009). Modeling the Global Fate and Transport
of Perfluorooctanoic Acid (PFOA) and Perfluorooctanoate (PFO) Emitted
from Direct Sources Using a Multispecies Mass Balance Model. Environ. Sci. Technol..

[5] Kurwadkar S., Dane J., Kanel S. R., Nadagouda M. N., Cawdrey R. W., Ambade B., Struckhoff G. C., Wilkin R. (2022). Per- and polyfluoroalkyl substances in water and wastewater:
A critical review of their global occurrence and distribution. Sci. Total Environ..

[6] Kato K., Wong L.-Y., Jia L. T., Kuklenyik Z., Calafat A. M. (2011). Trends in Exposure to Polyfluoroalkyl
Chemicals in
the U.S. Population: 1999–2008. Environ.
Sci. Technol..

[7] Calafat A.
M., Wong L.-Y., Kuklenyik Z., Reidy J. A., Needham L. L. (2007). Polyfluoroalkyl
Chemicals in the U.S. Population: Data from the National Health and
Nutrition Examination Survey (NHANES) 2003—2004 and Comparisons
with NHANES 1999—2000. Environ. Health
Perspect..

[8] Fenton S. E., Ducatman A., Boobis A., DeWitt J. C., Lau C., Ng C., Smith J. S., Roberts S. M. (2020). Per- and Polyfluoroalkyl Substance
Toxicity and Human Health Review: Current State of Knowledge and Strategies
for Informing Future Research. Environ. Toxicol.
Chem..

[9] O’Hagan, D. Understanding organofluorine chemistry. An introduction to the C–F bond Royal Society of Chemistry 37, pp 308–319. 10.1039/b711844a 18197347

[10] Raghavachari K., Trucks G. W., Pople J. A., Head-Gordon M. (1989). A fifth-order
perturbation comparison of electron correlation theories. Chem. Phys. Lett..

[11] Urban M., Noga J., Cole S. J., Bartlett R. J. (1985). Towards a full CCSDT
model for electron correlation. J. Chem. Phys..

[12] Watts J. D., Gauss J., Bartlett R. J. (1993). Coupled-cluster methods with noniterative
triple excitations for restricted open-shell Hartree–Fock and
other general single determinant reference functions. Energies and
analytical gradients. J. Chem. Phys..

[13] Theory and Applications of Computational Chemistry: The First Forty Years. 2005,.

[14] Frisch M. J., Head-Gordon M., Pople J. A. (1990). A direct MP2 gradient method. Chem. Phys. Lett..

[15] Kohn W., Sham L. J. (1965). Quantum Density
Oscillations in an Inhomogeneous Electron
Gas. Phys. Rev..

[16] Perdew J. P., Ruzsinszky A., Tao J., Staroverov V. N., Scuseria G. E., Csonka G. I. (2005). Prescription for the design and selection
of density functional approximations: More constraint satisfaction
with fewer fits. J. Chem. Phys..

[17] Mardirossian N., Head-Gordon M. (2017). Thirty years of density functional theory in computational
chemistry: an overview and extensive assessment of 200 density functionals. Mol. Phys..

[18] Israelachvili, J. N. Intermolecular and Surface Forces; Academic Press: Amsterdam; Boston, 2011.

[19] Omorodion H., Twamley B., Platts J. A., Baker R. J. (2015). Further Evidence
on the Importance of Fluorous–Fluorous Interactions in Supramolecular
Chemistry: A Combined Structural and Computational Study. Cryst. Growth Des..

[20] Kirsch, P. Modern Fluoroorganic Chemistry: Synthesis, Reactivity, Applications, 1st ed.; Wiley.

[21] Czajka A., Hazell G., Eastoe J. (2015). Surfactants at the
Design Limit. Langmuir.

[22] Van
Hoomissen D. J., Vyas S. (2019). Early Events in the Reductive Dehalogenation
of Linear Perfluoroalkyl Substances. Environ.
Sci. Technol. Lett..

[23] Hehre, W. J. ; Radom, L. ; Schleyer, P. v. R. ; Pople, J. A. Ab Initio Molecular Orbital Theory; Wiley-Interscience: New York, 1986; p 576.

[24] Radom L., Lathan W. A., Hehre W. J., Pople J. A. (1971). Molecular orbital
theory of the electronic structure of organic compounds. VIII. Geometries,
energies, and polarities of C3 hydrocarbons. J. Am. Chem. Soc..

[26] Ruscic B., Boggs J. E., Burcat A. (2005). Active Thermochemical
Tables (ATcT): An Open-Access Thermochemical Resource. J. Phys. Chem. Ref. Data.

[27] Ruscic, B. Active Thermochemical Tables (ATcT), Argonne National Laboratory. https://atct.anl.gov.

[28] Sharma S., Abeywardane K., Goldsmith C. F. (2023). Theory-Based
Mechanism for Fluoromethane
Combustion I: Thermochemistry and Abstraction Reactions. J. Phys. Chem. A.

[29] Somers K. P., Simmie J. M. (2015). Benchmarking Compound Methods (CBS-QB3, CBS-APNO, G3,
G4, W1BD) against the Active Thermochemical Tables: Formation Enthalpies
of Radicals. J. Phys. Chem. A.

[30] Buck R. C., Franklin J., Berger U., Conder J. M., Cousins I. T., de Voogt P., Jensen A. A., Kannan K., Mabury S. A., van Leeuwen S. P. (2015). Perfluoroalkyl and polyfluoroalkyl
substances in the
environment: Terminology, classification, and origins. Integr. Environ. Assess. Manage..

[31] Ram H., DePompa C. M., Westmoreland P. R. (2024). Thermochemistry of Gas-Phase Thermal
Oxidation of C2 to C8 Perfluorinated Sulfonic Acids with Extrapolation
to C16. J. Phys. Chem. A.

[32] Ram H., Sadej T. P., Murphy C. C., Mallo T. J., Westmoreland P. R. (2024). Thermochemistry
of Species in Gas-Phase Thermal Oxidation of C2 to C8 Perfluorinated
Carboxylic Acids. J. Phys. Chem. A.

[33] Abeywardane K., Goldsmith C. F. (2024). Accurate Enthalpies of Formation
for PFAS from First-Principles:
Combining Different Levels of Theory in a Generalized Thermochemical
Hierarchy. ACS Phys. Chem. Au.

[34] Grimme S., Ehrlich S., Goerigk L. (2011). Effect of
the damping function in
dispersion corrected density functional theory. J. Comput. Chem..

[35] Becke A. D. (1993). Density-functional
thermochemistry. III. The role of exact exchange. J. Chem. Phys..

[36] Lee C., Yang W., Parr R. G. (1988). Development of the Colle-Salvetti
correlation-energy formula into a functional of the electron density. Phys. Rev. B.

[37] Stephens P. J., Devlin F. J., Chabalowski C. F., Frisch M. J. (1994). Ab initio calculation
of vibrational absorption and circular dichroism spectra using density
functional force fields. J. Phys. Chem. A.

[38] Vosko S. H., Wilk L., Nusair M. (1980). Accurate spin-dependent
electron
liquid correlation energies for local spin density calculations: a
critical analysis. Can. J. Phys..

[39] Marenich A. V., Cramer C. J., Truhlar D. G. (2009). Universal
solvation model based on
solute electron density and on a continuum model of the solvent defined
by the bulk dielectric constant and atomic surface tensions. J. Phys. Chem. B.

[40] Bentel M. J., Yu Y., Xu L., Li Z., Wong B. M., Men Y., Liu J. (2019). Defluorination of Per- and Polyfluoroalkyl Substances (PFASs) with
Hydrated Electrons: Structural Dependence and Implications to PFAS
Remediation and Management. Environ. Sci. Technol..

[41] Linstrom, P. J. ; Mallard, W. G. NIST Chemistry WebBook, NIST Standard Reference Database Number 69; NIST, 2024, https://webbook.nist.gov/.

[42] Hait D., Head-Gordon M. (2018). How accurate is density functional
theory at predicting
dipole moments? An assessment using a new database of 200 benchmark
values. J. Chem. Theory Comput..

[43] Neese F., Wennmohs F., Becker U., Riplinger C. (2020). The ORCA Quantum
Chemistry Program Package. J. Chem. Phys..

[44] Frisch, M. J. ; Trucks, G. W. ; Schlegel, H. B. Gaussian 16, Revision A.03., Gaussian Inc 2016.

[45] Riplinger C., Neese F. (2013). An efficient
and near linear scaling pair natural orbital based local
coupled cluster method. J. Chem. Phys..

[46] Riplinger C., Sandhoefer B., Hansen A., Neese F. (2016). Natural triple excitations
in local coupled cluster calculations with pair natural orbitals. J. Chem. Phys..

[47] Kozuch S., Gruzman D., Martin J. M. L. (2010). DSD-BLYP:
A General Purpose Double
Hybrid Density Functional Including Spin Component Scaling and Dispersion
Correction. J. Phys. Chem. C.

[48] Perdew J. P., Wang Y. (1992). Accurate and simple analytic representation of the electron-gas correlation
energy. Phys. Rev. B.

[50] Handy N. C., Cohen A. J. (2001). Left-right correlation
energy. Mol. Phys..

[51] Hamprecht F. A., Cohen A. J., Tozer D. J., Handy N. C. (1998). Development and
assessment of new exchange-correlation functionals. J. Chem. Phys..

[52] Zhang Y., Yang W. (1998). Comment on “Generalized Gradient Approximation Made Simple. Phys. Rev. Lett..

[53] Staroverov V. N., Scuseria G. E., Tao J., Perdew J. P. (2003). Comparative
assessment
of a new nonempirical density functional: Molecules and hydrogen-bonded
complexes. J. Chem. Phys..

[54] Zhao Y., Truhlar D. G. (2005). Design of Density
Functionals That Are Broadly Accurate
for Thermochemistry, Thermochemical Kinetics, and Nonbonded Interactions. J. Phys. Chem. A.

[55] Sun J., Ruzsinszky A., Perdew J. P. (2015). Strongly Constrained and Appropriately
Normed Semilocal Density Functional. Phys. Rev.
Lett..

[56] Mardirossian N., Head-Gordon M. (2014). *ω*B97X-V: A 10-parameter, range-separated
hybrid, generalized gradient approximation density functional with
nonlocal correlation. J. Chem. Phys..

[57] Brémond E., Adamo C. (2011). Seeking for parameter-free
double-hybrid functionals: The PBE0-DH
model. J. Chem. Phys..

[58] Peverati R., Truhlar D. G. (2012). Screened-Exchange Density Functionals with Broad Accuracy
for Chemistry and Solid-State Physics. J. Chem.
Theory Comput..

[59] Chai J.-D., Head-Gordon M. (2008). Long-range
corrected hybrid density functionals with
damped atom–atom dispersion corrections. Phys. Chem. Chem. Phys..

[60] Grimme S. (2006). Semiempirical
hybrid density functional with perturbative second-order correlation. J. Chem. Phys..

[61] Mardirossian N., Head-Gordon M. (2016). *ω*B97M-V: A combinatorially optimized,
range-separated hybrid, meta-GGA density functional with VV10 nonlocal
correlation. J. Chem. Phys..

[62] Grimme S. (2006). Semiempirical
GGA-type density functional constructed with a long-range dispersion
correction. J. Comput. Chem..

[63] Mardirossian N., Head-Gordon M. (2015). Mapping the
genome of meta-generalized gradient approximation
density functionals: The search for B97M-V. J. Chem. Phys..

[64] Grimme S., Brandenburg J. G., Bannwarth C., Hansen A. (2015). Consistent structures
and interactions by density functional theory with small atomic orbital
basis sets. J. Chem. Phys..

[65] Ehlert S., Grimme S., DiStasio R. A. (2021). r^2^SCAN-3c: A “Swiss Army Knife” composite
electronic-structure method. J. Chem. Phys..

[66] Bannwarth C., Ehlert S., Grimme S. (2019). GFN2-xTB–An
Accurate and Broadly
Parametrized Self-Consistent Tight-Binding Quantum Chemical Method
with Multipole Electrostatics and Density-Dependent Dispersion Contributions. J. Chem. Theory Comput..

[67] Goldsmith C. F., Magoon G. R., Green W. H. (2012). Database of Small
Molecule Thermochemistry
for Combustion. J. Phys. Chem. A.

[68] Klippenstein S. J., Harding L. B. (2009). Kinetics of the H+NCO reaction. Proc. Combust. Inst..

[69] Yu H., Song S., Nam S., Burke K., Sim E. (2023). Density-Corrected
Density Functional Theory for Open Shells: How to Deal with Spin Contamination. J. Phys. Chem. Lett..

[70] Williams T. G., DeYonker N. J., Ho B. S., Wilson A. K. (2011). The correlation
Consistent composite Approach: The spin contamination effect on an
MP2-based composite methodology. Chem. Phys.
Lett..

[71] Rettig A., Hait D., Bertels L. W., Head-Gordon M. (2020). Third-Order
Møller–Plesset Theory Made More Useful? The Role of Density
Functional Theory Orbitals. J. Chem. Theory
Comput..

[72] Purvis G. D., Bartlett R. J. (1982). A full coupled-cluster
singles and doubles model: The
inclusion of disconnected triples. J. Chem.
Phys..

[74] Guo Y., Riplinger C., Becker U., Liakos D. G., Neese F. (2018). Communication:
An improved linear scaling perturbative triples correction for the
domain based local pair-natural orbital based coupled cluster singles
and doubles method [DLPNO-CCSD­(T)]. J. Chem.
Phys..

[75] Montgomery J. A., Frisch M. J., Ochterski J. W., Petersson G. A. (1999). A complete
basis set model chemistry. VI. Use of density functional geometries
and frequencies. J. Chem. Phys..

[76] Petersson G. A., Bennett A., Tensfeldt T. G., Al-Laham M. A., Shirley W. A., Mantzaris J. (1988). A complete
basis set model chemistry. I. The total
energies of closed-shell atoms and hydrides of the first-row elements. J. Chem. Phys..

[77] Barnes E.
C., Petersson G. A., Montgomery J. A. J., Frisch M. J., Martin J. M. L. (2009). Unrestricted
Coupled Cluster and Brueckner Doubles Variations of W1 Theory. J. Chem. Theory Comput..

[78] Martin J. M. L., de Oliveira G. (1999). Towards standard methods for benchmark quality ab initio
thermochemistry—W1 and W2 theory. J.
Chem. Phys..

[79] Curtiss L.
A., Redfern P. C., Raghavachari K., Rassolov V., Pople J. A. (1999). Gaussian-3
theory using reduced Møller–Plesset order. J. Chem. Phys..

[80] Curtiss L.
A., Redfern P. C., Raghavachari K. (2007). Gaussian-4 theory. J. Chem. Phys..

[81] Møller C., Plesset M. S. (1934). Note on an Approximation Treatment for Many-Electron
Systems. Phys. Rev..

[200] Chai J.-D., Head-Gordon M. (2009). Long-Range
Corrected Double-Hybrid
Density Functionals. J. Chem. Phys..

[82] Peverati R., Truhlar D. G. (2011). Improving the Accuracy
of Hybrid Meta-GGA Density Functionals
by Range Separation. J. Phys. Chem. Lett..

[83] Lin Y.-S., Li G.-D., Mao S.-P., Chai J.-D. (2013). Long-Range Corrected
Hybrid Density Functionals with Improved Dispersion Corrections. J. Chem. Theory Comput..

[84] Yanai T., Tew D. P., Handy N. C. (2004). A new hybrid
exchange–correlation
functional using the Coulomb-attenuating method (CAM-B3LYP). Chem. Phys. Lett..

[85] Zhao Y., Truhlar D. G. (2008). The M06 suite of density functionals
for main group
thermochemistry, thermochemical kinetics, noncovalent interactions,
excited states, and transition elements. Theor.
Chem. Acc..

[86] Yu H. S., He X., Li S. L., Truhlar D. G. (2016). MN15: A Kohn–Sham global-hybrid
exchange–correlation density functional with broad accuracy
for multi-reference and single-reference systems and noncovalent interactions. Chem. Sci..

[91] Adamo C., Barone V. (1999). Toward reliable density functional methods without
adjustable parameters: The PBE0 model. J. Chem.
Phys..

[92] Peverati R., Truhlar D. G. (2012). M11-L: A local density
functional for electronic structure
calculations of molecules and condensed phases. J. Phys. Chem. Lett..

[93] Perdew J. P., Burke K., Ernzerhof M. (1996). Generalized Gradient Approximation
Made Simple. Phys. Rev. Lett..

[94] Becke A. D. (1988). Density-functional
exchange-energy approximation with correct asymptotic behavior. Phys. Rev. A.

[95] Perdew J. P. (1986). Density-functional
approximation for the correlation energy of the inhomogeneous electron
gas. Phys. Rev. B.

[96] Roothaan C. C. J. (1951). New
Developments in Molecular Orbital Theory. Rev.
Mod. Phys..

[97] Stewart J. J. P. (2013). Optimization
of parameters for semiempirical methods VI: More modifications to
the NDDO approximations and re-optimization of parameters. J. Mol. Model..

[98] Li Y.-P., Gomes J., Sharada S. M., Bell A. T., Head-Gordon M. (2015). Improved Force-Field
Parameters for QM/MM Simulations of the Energies of Adsorption for
Molecules in Zeolites and a Free Rotor Correction to the Rigid Rotor
Harmonic Oscillator Model for Adsorption Enthalpies. J. Phys. Chem. C.

[99] Xu S., Wang Q.-D., Sun M.-M., Yin G., Liang J. (2021). Benchmark
calculations for bond dissociation energies and enthalpy of formation
of chlorinated and brominated polycyclic aromatic hydrocarbons. RSC Adv..

[100] Jiang L., Xu Y., Zhang X., Xu B., Xu X., Ma Y. (2022). Developing
a QSPR Model of Organic Carbon Normalized
Sorption Coefficients of Perfluorinated and Polyfluoroalkyl Substances. Molecules.

[101] Zapata J. C., McKemmish L. K. (2020). Computation of Dipole Moments: A
Recommendation on the Choice of the Basis Set and the Level of Theory. J. Phys. Chem. A.

[102] Soyemi A., Szilvásí T. (2022). Benchmarking Semiempirical
QM Methods for Calculating the Dipole Moment of Organic Molecules. J. Phys. Chem. A.

[103] Ásgeirsson V., Bauer C. A., Grimme S. (2017). Quantum chemical
calculation
of electron ionization mass spectra for general organic and inorganic
molecules. Chem. Sci..

[104] North S. C., Jorgensen K. R., Pricetolstoy J., Wilson A. K. (2023). Population analysis and the effects
of Gaussian basis
set quality and quantum mechanical approach: main group through heavy
element species. Front. Chem..

[105] Cramer, C. J. Essentials of Computational Chemistry: Theories and Models, 2nd ed.; John Wiley & Sons: Chichester, UK, 2013; p 315.

[106] Mulliken R. S. (1955). Electronic Population Analysis on LCAO-MO Molecular
Wave Functions. I. J. Chem. Phys..

[107] Szabo, A. ; Ostlund, N. S. Modern Quantum Chemistry: Introduction to Advanced Electronic Structure Theory; Dover Publications, 1989.

[108] Weinhold, F. ; Carpenter, J. E. The Structure of Small Molecules and Ions; Naaman, R. ; Vager, Z. , Eds.; Plenum Press: New York, 1988; pp 227–236.

