# Supplementary material for: A Comprehensive Benchmark Database of Per- and Polyfluoroalkyl Substance Properties from Quantum Mechanical Methods
Source: J Chem Inf Model. 2026 Apr 29;66(9):5287–300. doi: 10.1021/acs.jcim.5c03092 (PMC13169373; doi:10.1021/acs.jcim.5c03092)
Supplement: Supplementary file 1 [file ci5c03092_si_001.pdf]

Supporting Information: A Comprehensive Benchmark Database of Per- and Polyfluoroalkyl Substance Properties from Quantum Mechanical Methods

Melissa Marciesky,† Scott Simpson,‡ John Keith,*,† and Carla Ng*,¶,†

†*Department of Chemical and Petroleum Engineering, University of Pittsburgh, Pittsburgh, PA, USA*

‡*Department of Chemistry, St. Bonaventure University, Buffalo, NY, USA*

¶*Department of Civil and Environmental Engineering, University of Pittsburgh, Pittsburgh, PA, USA*

E-mail: jakeith@pitt.edu; carla.ng@pitt.edu

Full statistical analysis values (Tables S2–S21) are provided in the accompanying "Comprehensive_Benchmark_PFAS_SI2.xlsb" file. Individual molecular properties by method are available in the database, and corresponding experimental values are listed in Table S21. The complete set of database .csv files can be accessed at github.com/Ng-Lab-Group/PFAS_Database. Bond dissociation energies (BDEs) can be computed manually from the individual molecular enthalpies in the database; however, an automated helper script is available at github.com/Ng-Lab-Group/PFAS_BDE_helper.

Supporting Information: Table of Contents

A Comprehensive Benchmark Database of Per- and Polyfluoroalkyl Substance Properties from Quantum Mechanical Methods

- **Section S1. Thermodynamic Properties**
  - **S1.1 Basis Set Comparison** .... Figure S1, Table S1

    Basis set performance comparison and representative wall-time example.
  - **S1.2 Experimental BDE Comparison** .... Figure S2

    Benchmarking computed BDEs against available experimental values.
  - **S1.3 BDE Performance Relative to Theory** .... Figures S3–S4

    Error analysis across methods for BDE.

- **Section S2. Electronic Properties**
  - **S2.1 Dipole Moment Basis Set Analysis** .... Figure S5

    Basis set dependence of computed dipole moments.
  - **S2.2 Experimental Dipole Moment Comparison** .... Figure S6

    Comparison of computed dipole moments with experimental data.
  - **S2.3 Dipole Moment Performance Relative to Theory** .. Figures S7–S11

    Error analysis across methods for dipole moments.
  - **S2.4 Electron Affinity and Ionization Potential: Experimental** . Figures S12–S13

    Comparison of computed EA and IP values with experimental measurements.
  - **S2.5 Ionization Potential Performance Relative to Theory** ...... Figures S14–S16

    Error analysis for IP across methods.

– **S2.6 Electron Affinity Performance Relative to Theory** Figures S17–S19

Error analysis for EA across methods.

Thermodynamic Properties

Basis Set Comparison

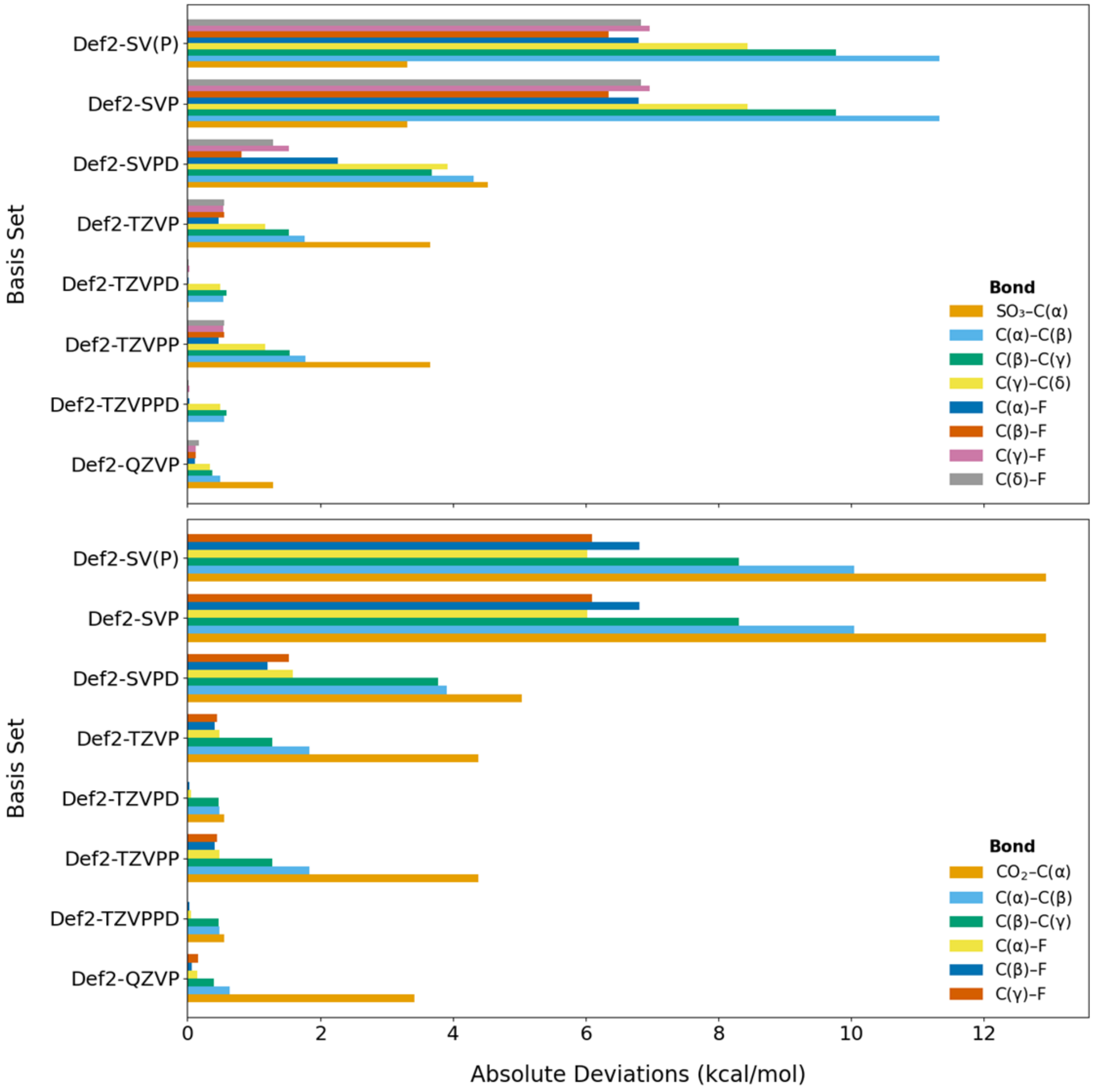

Figure S1: Basis set dependence of BDEs for PFBS (top) and PFBA (bottom) in deprotonated forms using the Karlsruhe Def2 series as compared to def2-QZVPD. The Def2-TZVPD basis set is the recommended level of theory for its efficiency and accuracy.

The table S1 below shows the CPU time for the anionic radical CO_2^-–$CF_2CF_2CF_2^*$. Frequency calculations were performed using B3LYP with 35 of memory.

Table S1: Comparison of frequency calculation walltimes (in hours) for different basis sets.

Basis Set	Def2-SV(P)	Def2-SVP	Def2-SVPD	Def2-TZVP	Def2-TZVPD	Def2-TZVPP	Def2-TZVPPD	Def2-QZVP	Def2-QZVPD
CPU time for frequency calculation (hours)	0.087	0.085	0.272	1.279	1.885	0.818	1.930	8.110	36.710

Experimental BDE Comparison

The experimental statistics of bond dissociation energies for all completed cases (n=8). For systems where certain BDEs could not be obtain due to feasibility the corresponding value of n is indicated above the bar.

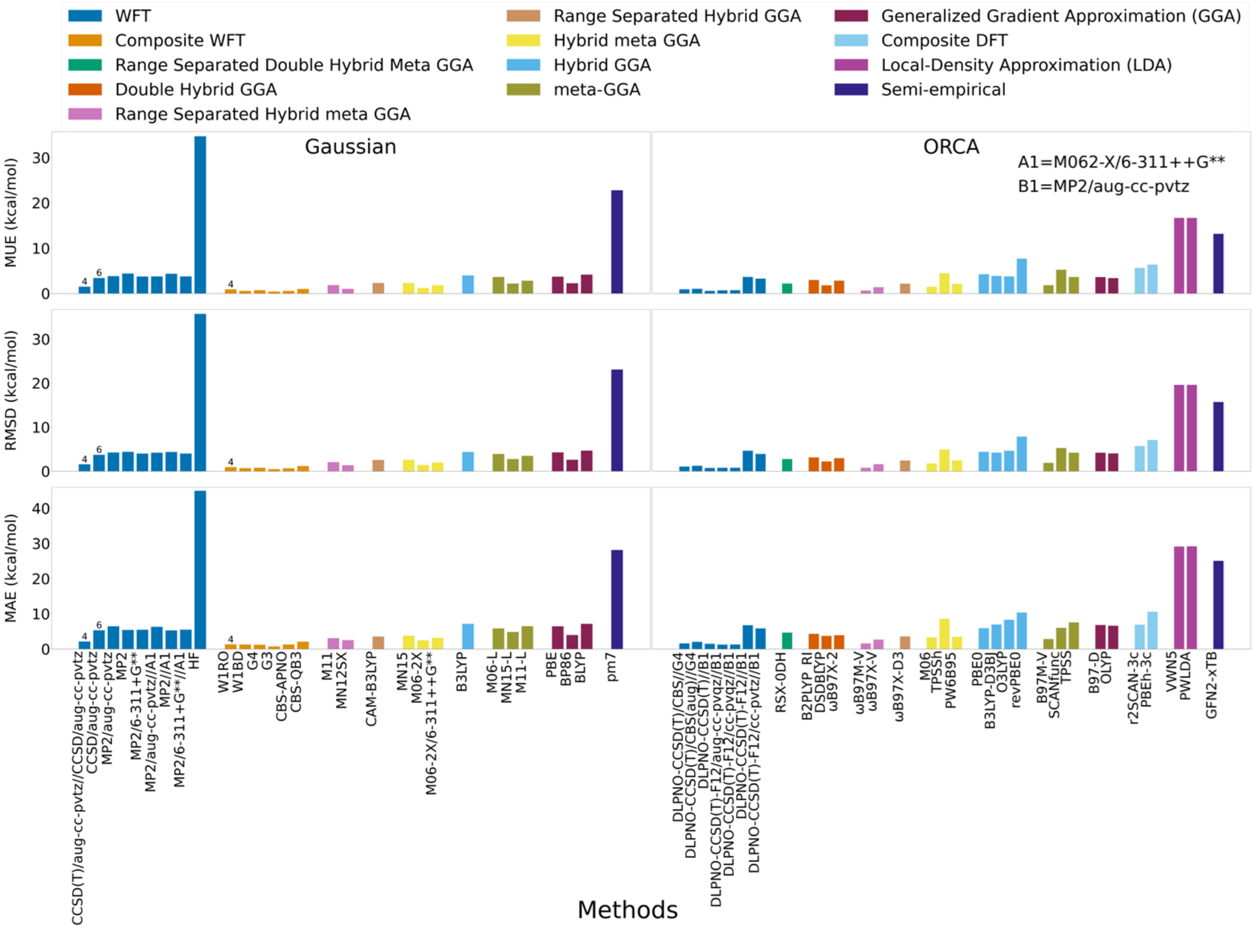

Figure S2: The MUE, RMSD, and MAE of BDEs for all methods used in this study against experimental BDE points (n=8). For systems where certain BDEs could not be obtained the corresponding value of n is indicated above the bar.

BDE Performance Relative to Theory

The statistics of bond dissociation energies for all completed cases (n=87) for neutral PFAS and PFAS-like molecules relative to G4. For systems where certain BDEs could not be obtain due the corresponding value of n is indicated above the bar.

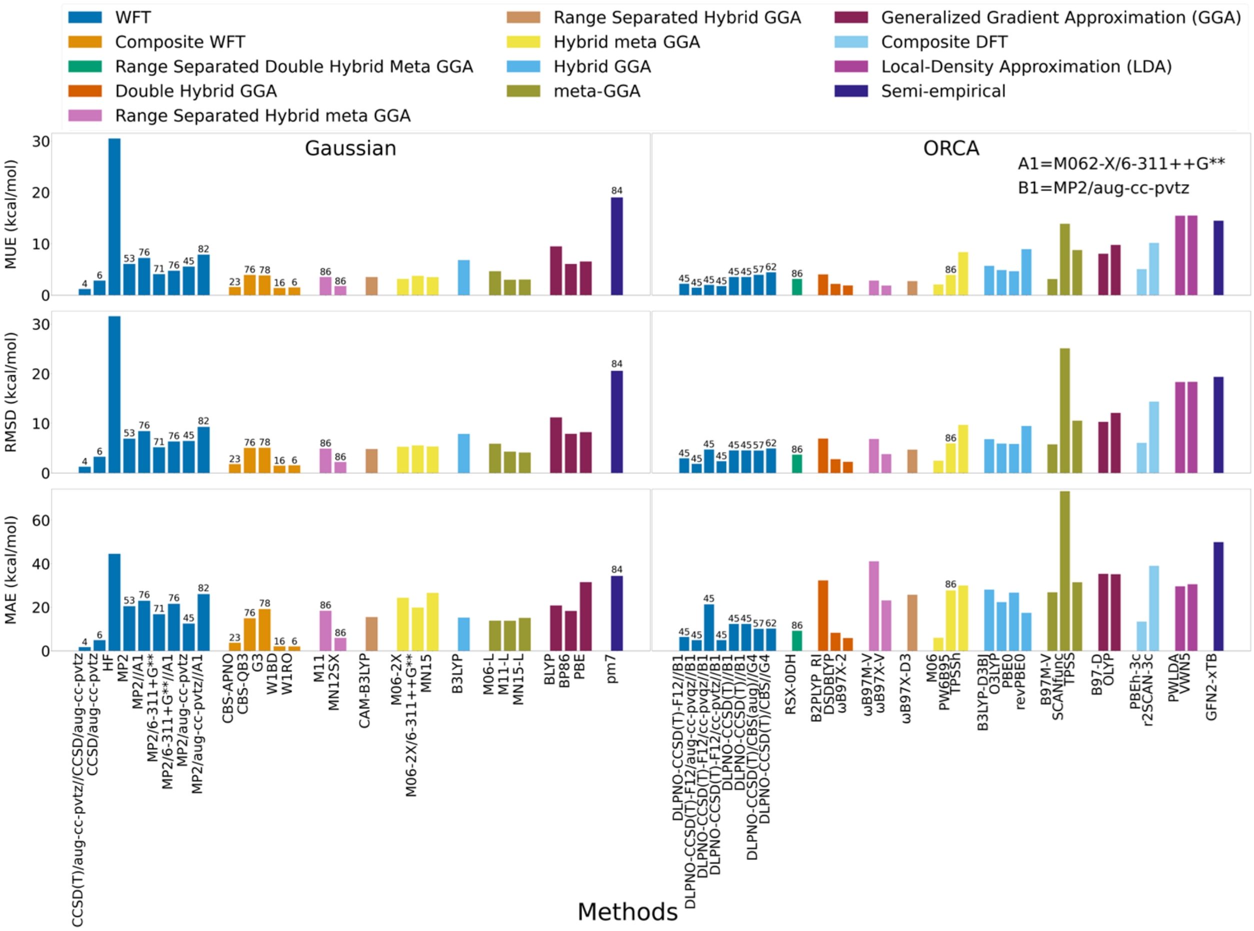

Figure S3: The MUE, RMSD, and max absolute error of BDEs for all methods used in this study as compared to G4 BDE points (n=87) for neutral PFAS. For systems where certain BDEs are not obtained the corresponding value of n is indicated above the bar.

The statistics of bond dissociation energies for all completed cases (n=43) for anionic PFAS and PFAS-like molecules relative to G4. For systems where certain BDEs could not be obtain due the corresponding value of n is indicated above the bar.

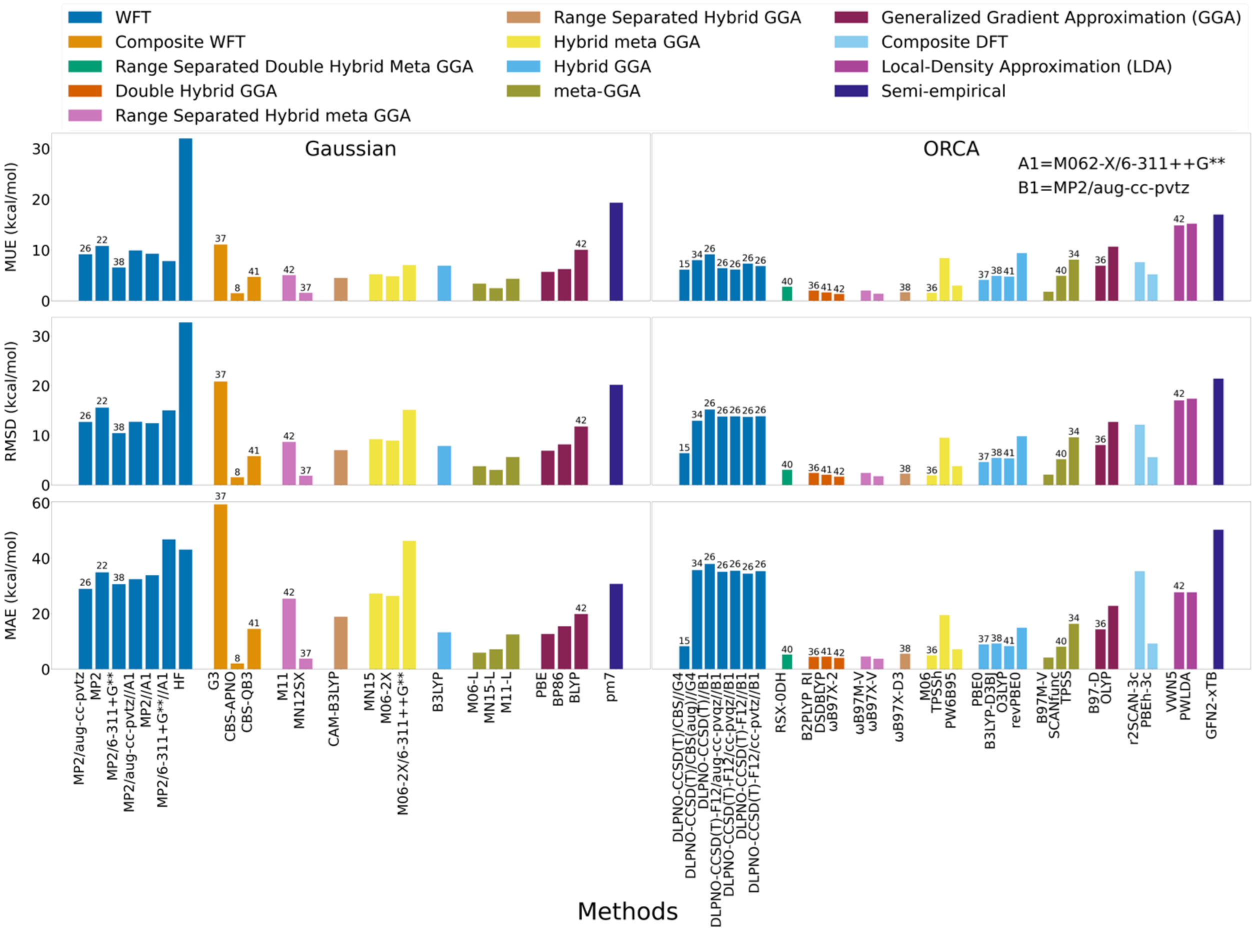

Figure S4: The MUE, RMSD, and max absolute error of BDEs for all methods used in this study as compared to G4 BDE points (n=43) for anionic PFAS. For systems where certain BDEs are not obtained the corresponding value of n is indicated above the bar.

Electronic Properties

Dipole Moment Basis Set Analysis

The basis set analysis of 43 PFAS and PFAS-like molecules for dipole moment done using M06-2X compared to def2-QZVPD.

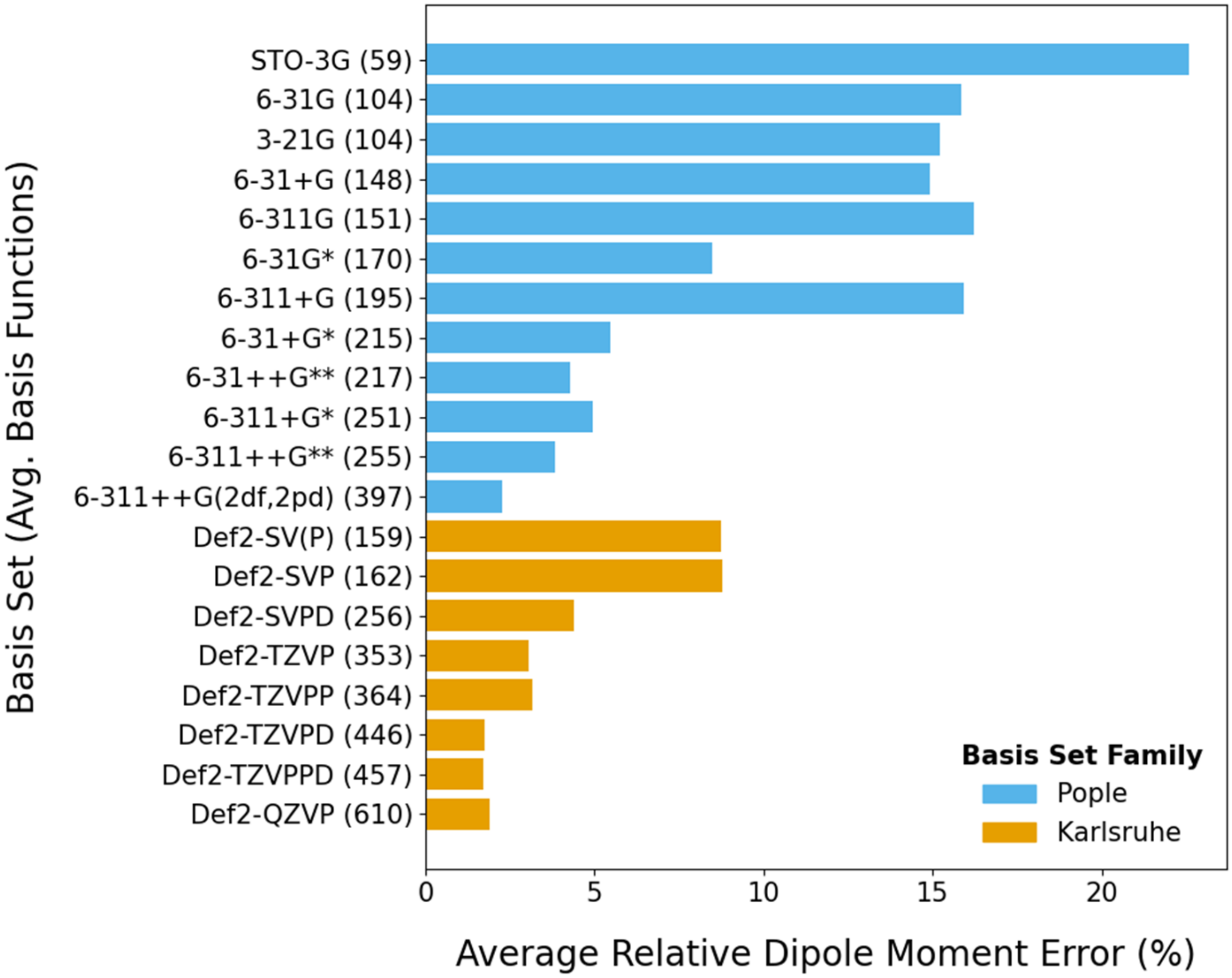

Figure S5: Average relative dipole moment error to def2-QZVPD for anionic PFAS and PFAS-like molecules using M06-2X (n=43). The basis set chosen from this evaluation was def2-TZVPD.

Experimental Dipole Moment Comparison

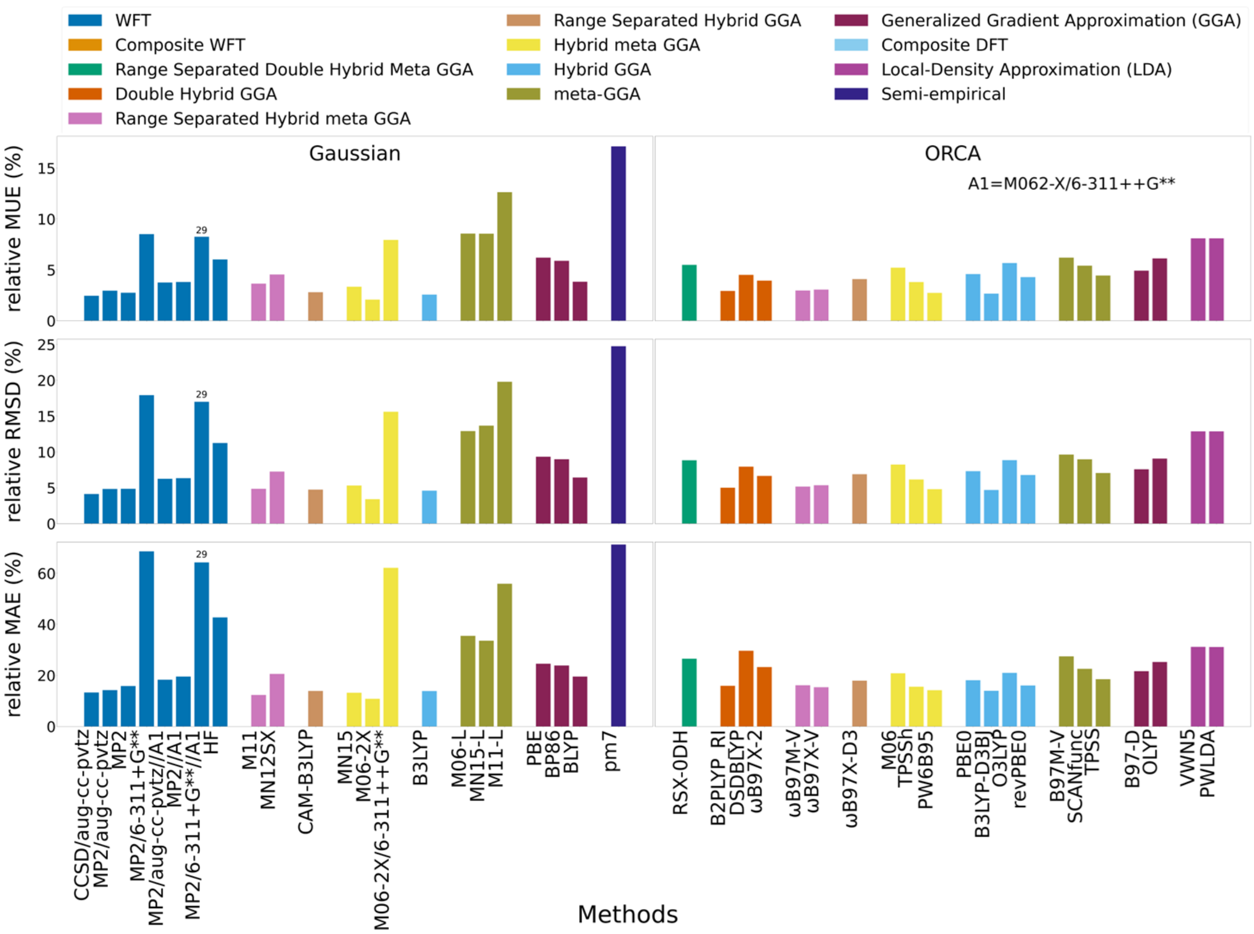

Figure S6: The relative MUE, RMSD, and maximum absolute error dipole moments across PFAS-like molecules relative to experimental data and the Hait and Head-Gordon data set.(n=31)

Dipole Moment Performance Relative to Theory

The statistics for relative dipole moment for all completed cases (n=77) relative to M06-2X/Def2-TZVPD for Neutral closed- and open-shelled PFAS.

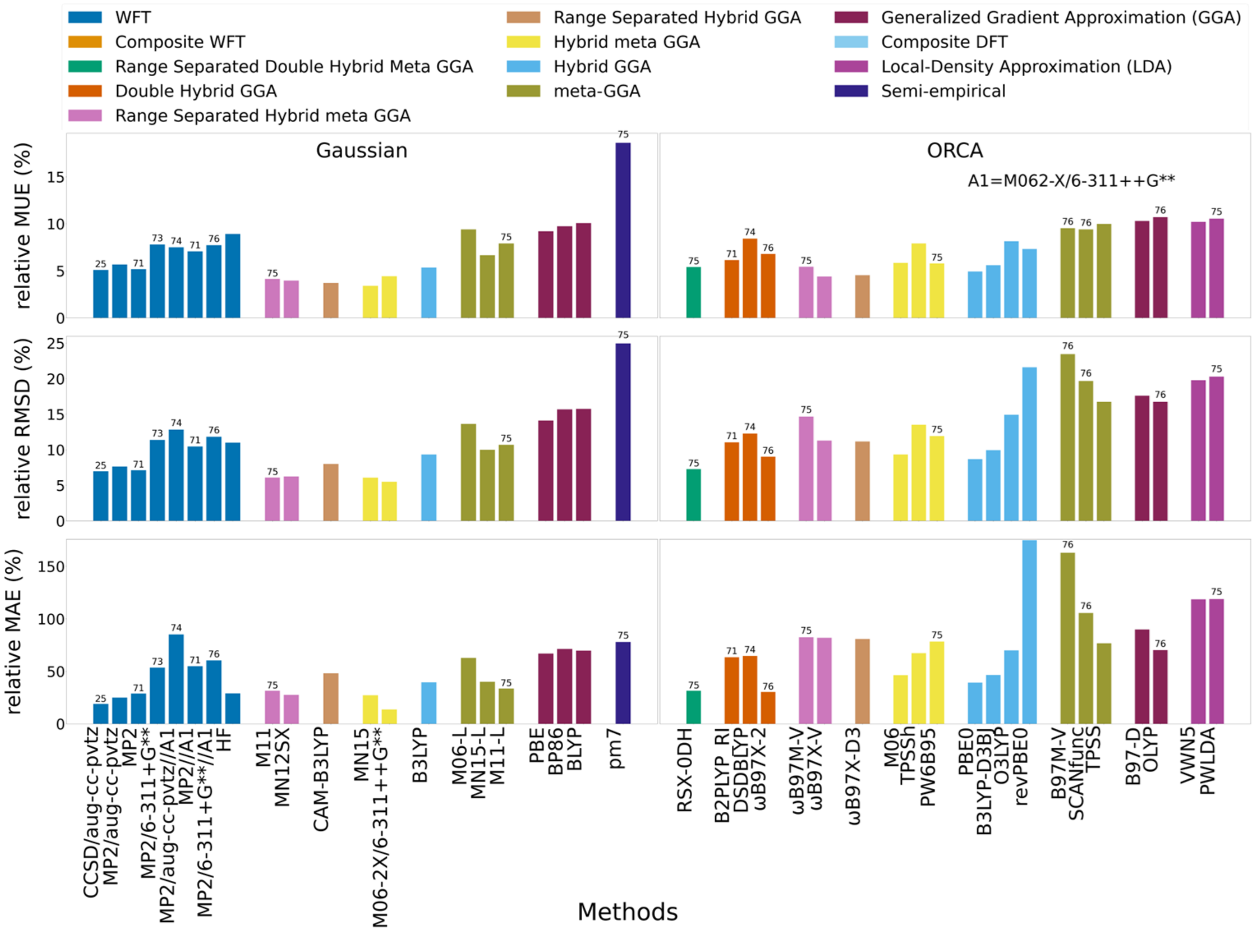

Figure S7: The relative MUE, RMSD, and max absolute error for dipole moments for all methods used in this study as compared to M06-2X/Def2-TZVPD points (n=77) for Neutral PFAS. For systems where certain BDEs are not obtained the corresponding value of n is indicated above the bar.

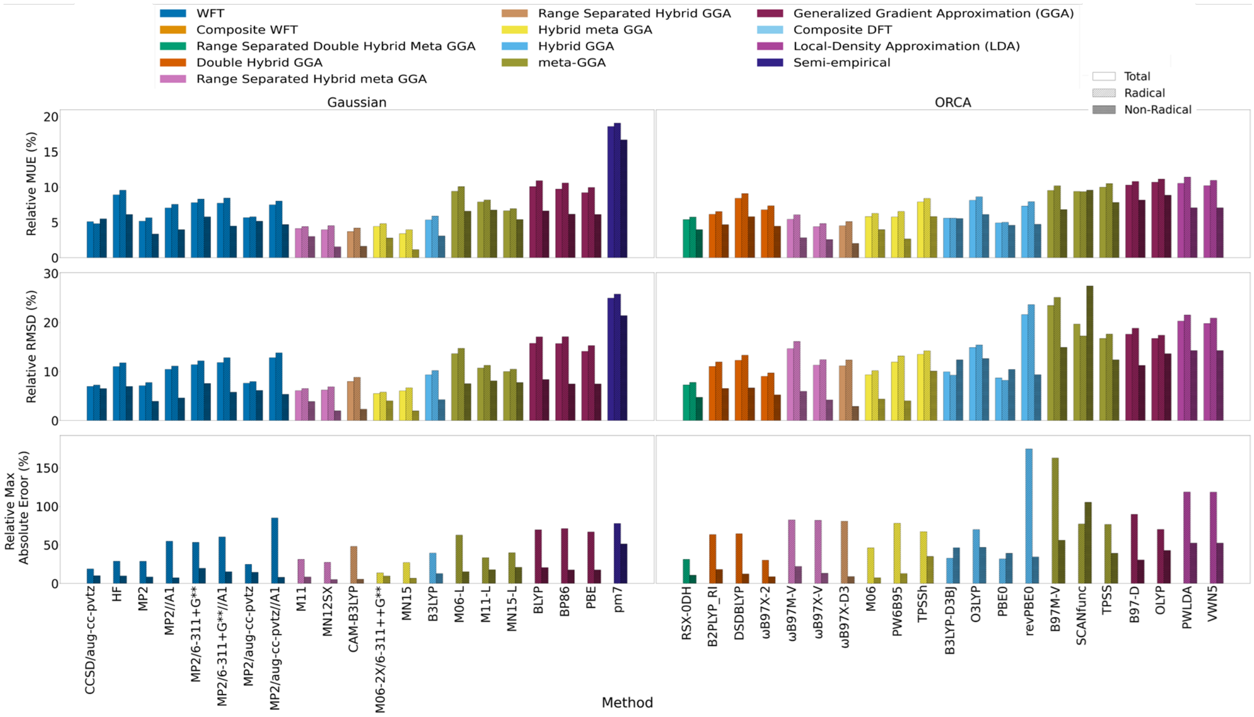

Figure S8: The relative MUE, RMSD, and max absolute error for dipole moments for all methods used in this study as compared to M06-2X/Def2-TZVPD points (n=77) for neutral PFAS broken into radical versus non radical molecules.

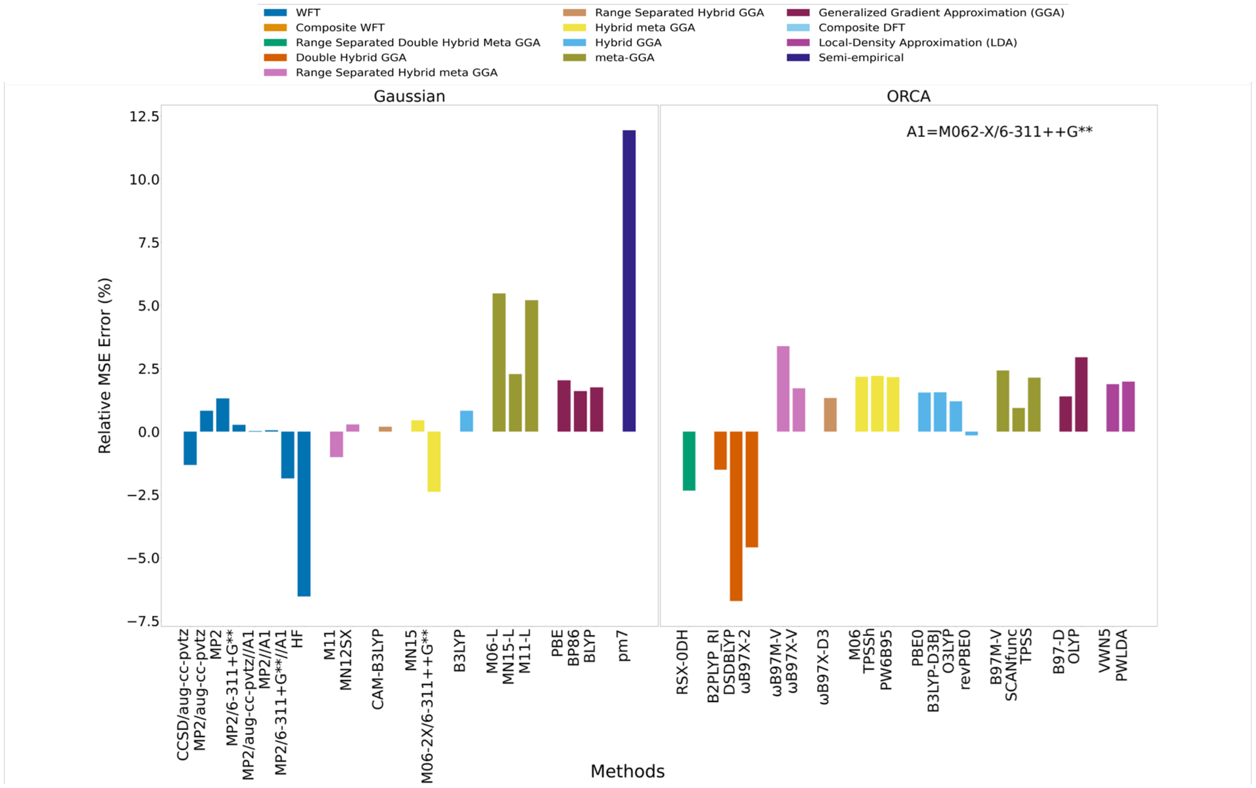

Figure S9: The relative MSE for dipole moments for all methods used in this study as compared to M06-2X/Def2-TZVPD points (n=77) for neutral/protonated PFAS.

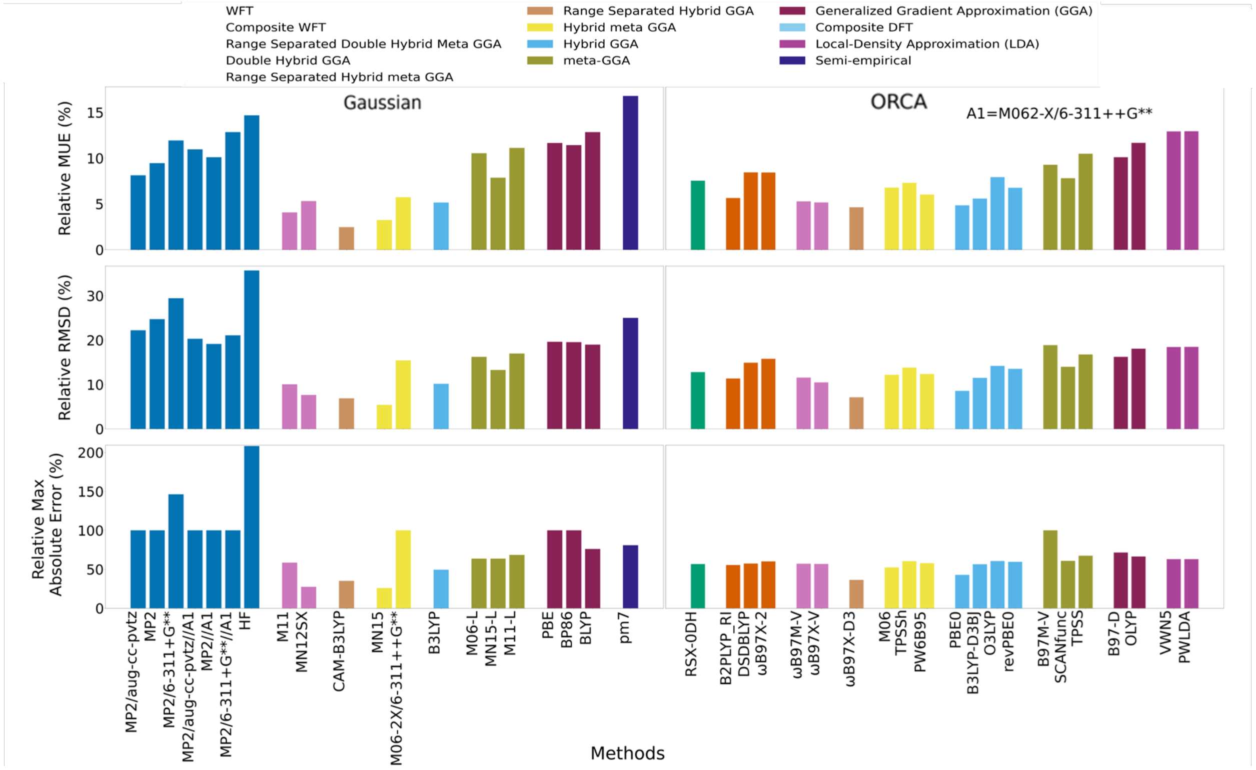

Figure S10: The relative MUE, RMSD, and max absolute error for dipole moments for all methods used in this study as compared to M06-2X/Def2-TZVPD points (n=51) for anionic/deprotonated PFAS. For systems where certain dipole moments are not obtained the corresponding value of n is indicated above the bar.

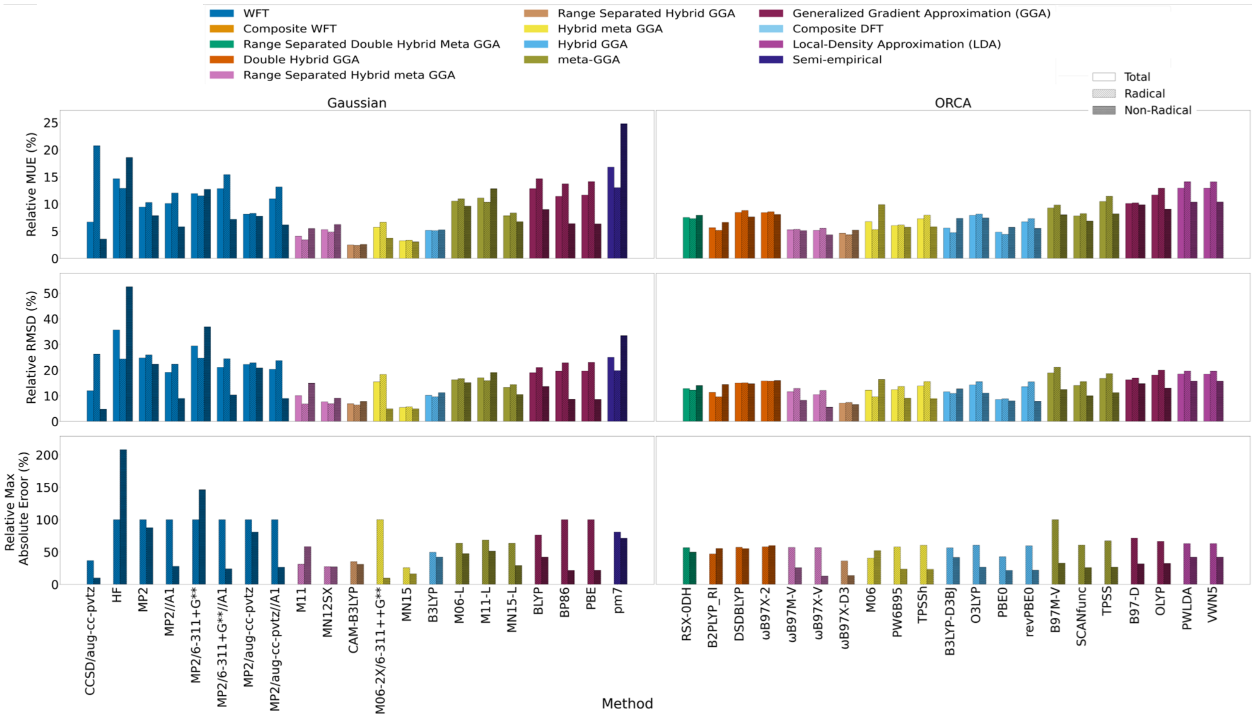

Figure S11: The relative MUE, RMSD, and max absolute error for dipole moments for all methods used in this study as compared to M06-2X/Def2-TZVPD points (n=51) for anionic/deprotonated PFAS broken into radical and non radical molecules.

Electron Affinity and Ionization Potential: Experimental

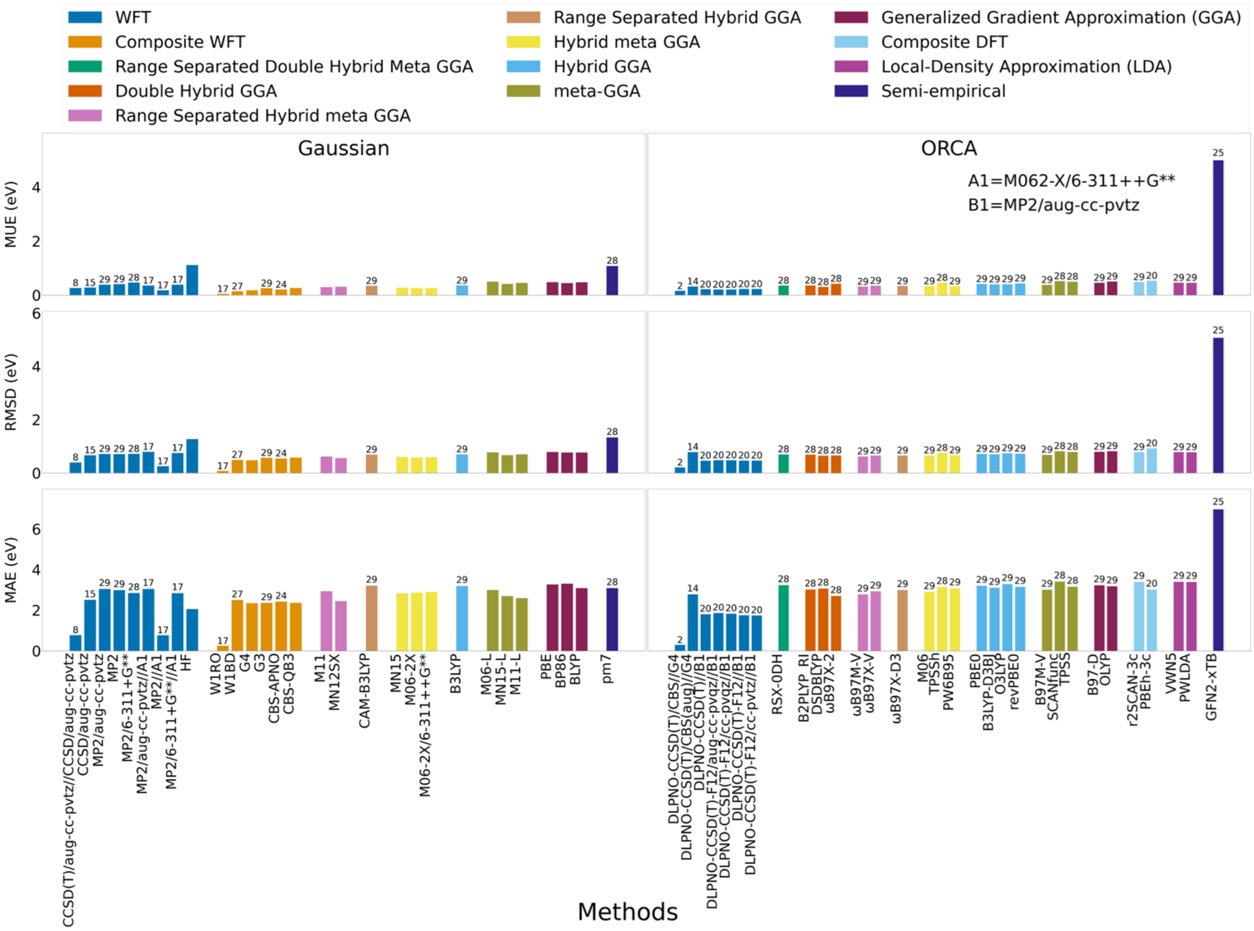

Figure S12: The MUE, RMSD, and MAE of ionization potential (IP) for all methods used in this study against experimental IP points (n=30). For systems where certain IPs could not be obtained the corresponding value of n is indicated above the bar.

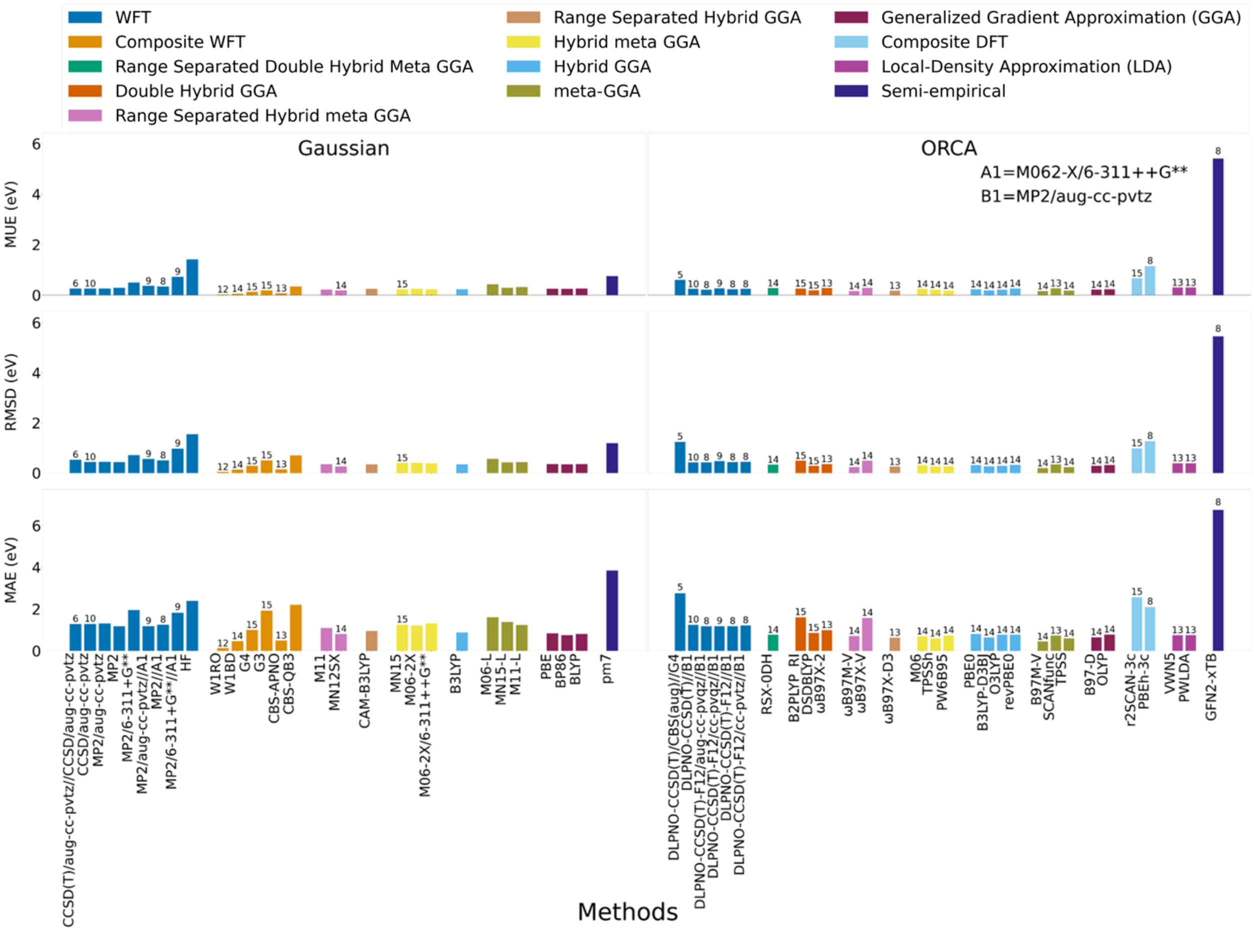

Figure S13: The MUE, RMSD, and max absolute error of electron affinity (EA) for all methods used in this study against experimental EA points (n=16). For systems where certain EAs could not be obtained the corresponding value of n is indicated above the bar.

Ionization Potential Performance Relative to Theory

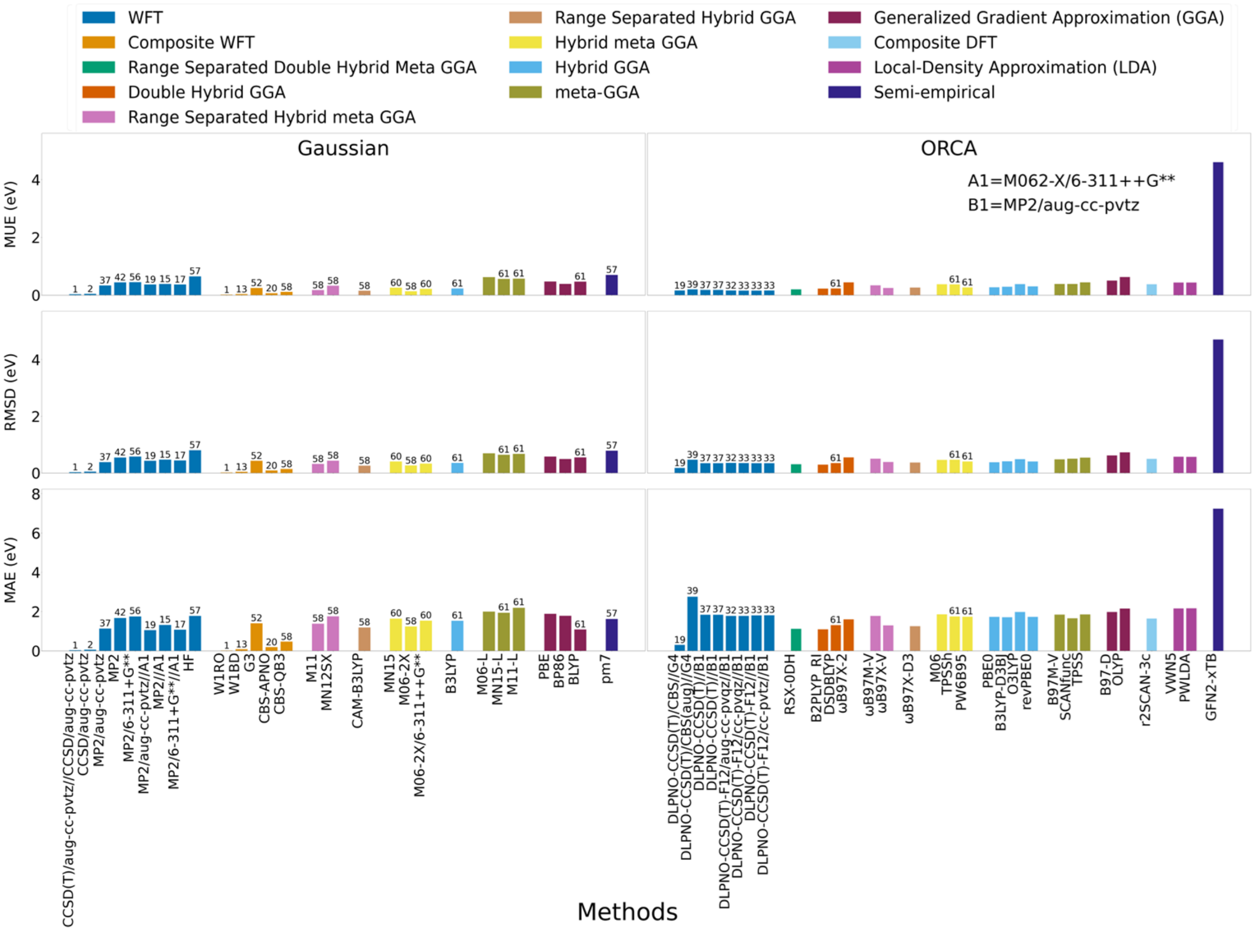

Figure S14: The MUE, RMSD, and max absolute error of ionization potential (IP) for all methods used in this study against G4 IP points (n=62) for neutral and neutral radical PFAS and PFAS-like molecules. For systems where certain IPs could not be obtained the corresponding value of n is indicated above the bar.

The MUE, RMSD, and max absolute error of ionization potential (IP) for all methods used in this study against G4 split into radical and non radical molecules for neutral and neutral radical PFAS and PFAS-like molecules. This entire 62 set contains 16 nuetral and 46 neutral radical molecules.

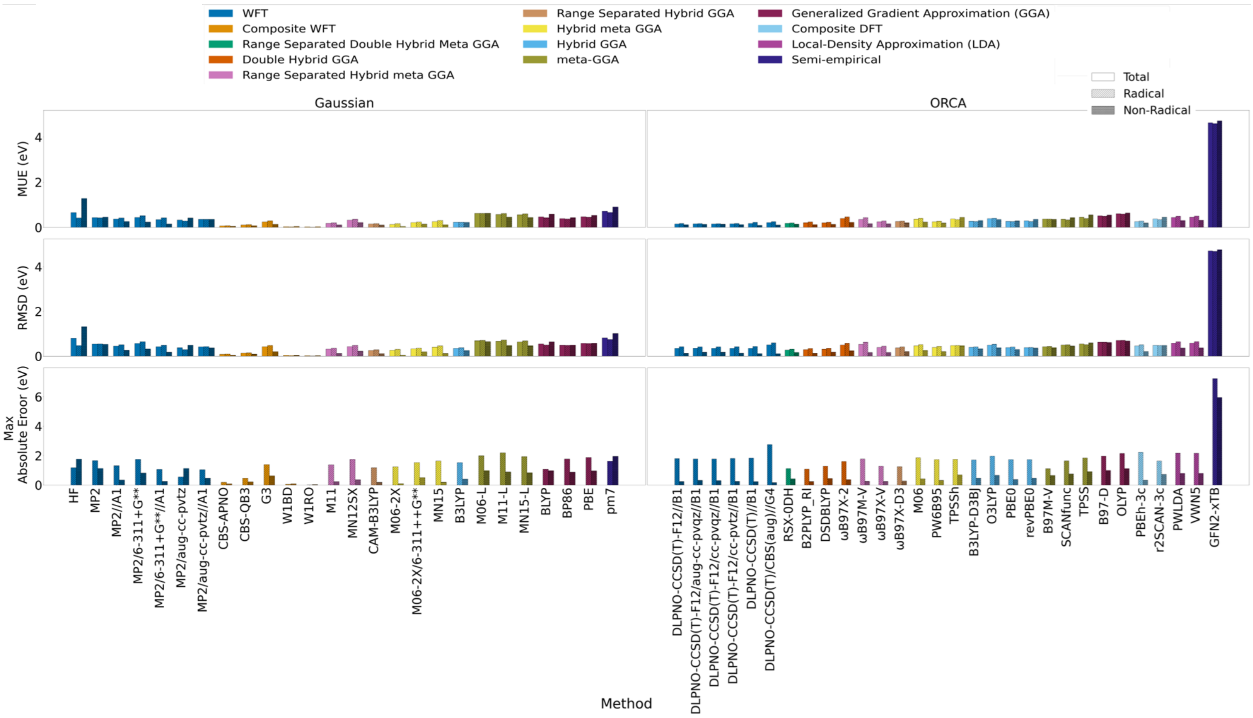

Figure S15: The MUE, RMSD, and max absolute error of ionization potential (IP) for all methods used in this study against G4 split into radical and non radical molecules for neutral and neutral radical PFAS and PFAS-like molecules.

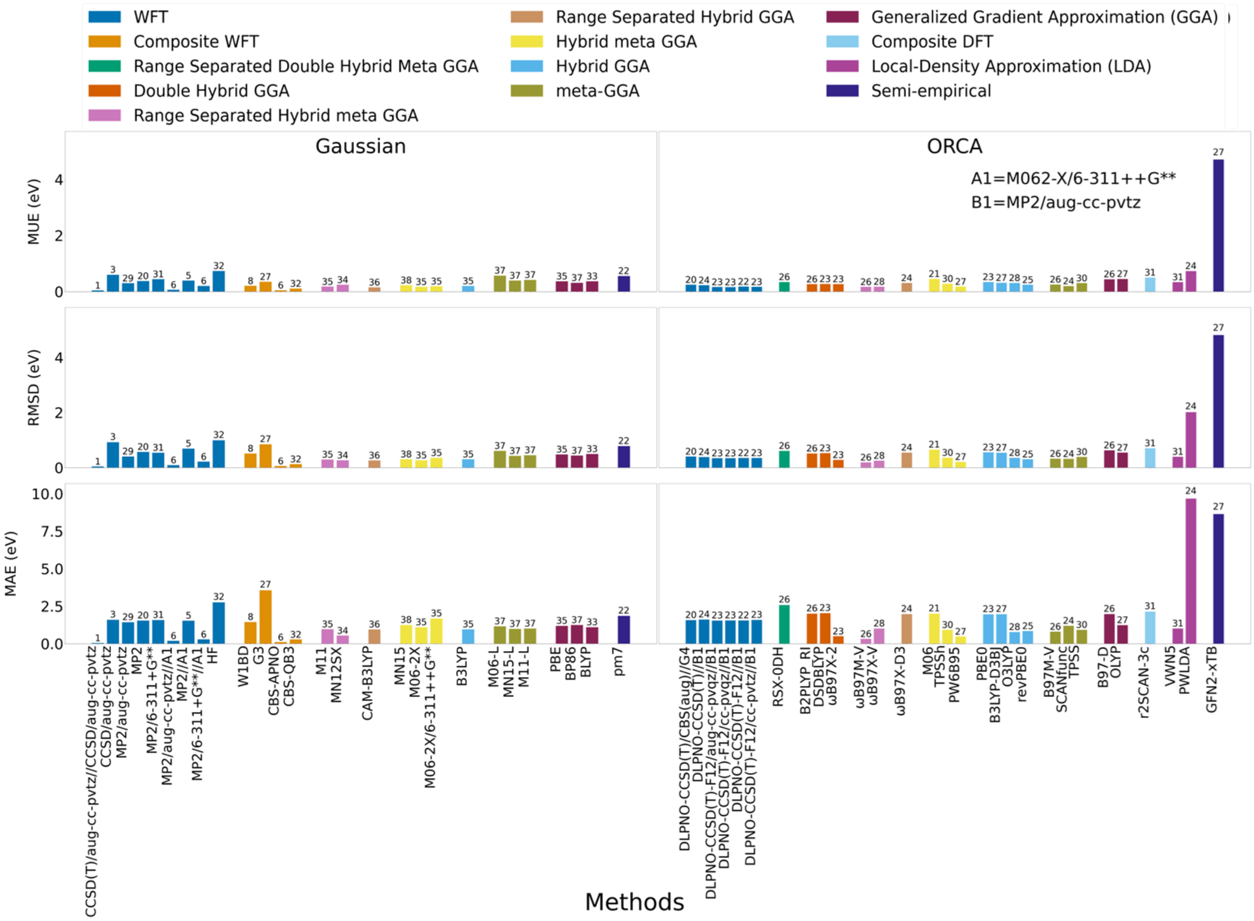

Figure S16: The MUE, RMSD, and max absolute error of ionization potential (IP) for all methods used in this study against G4 IP points (n=39) for anionic and anionic radical PFAS and PFAS-like molecules. For systems where certain IPs could not be obtained the corresponding value of n is indicated above the bar.

Electron Affinity Performance Relative to Theory

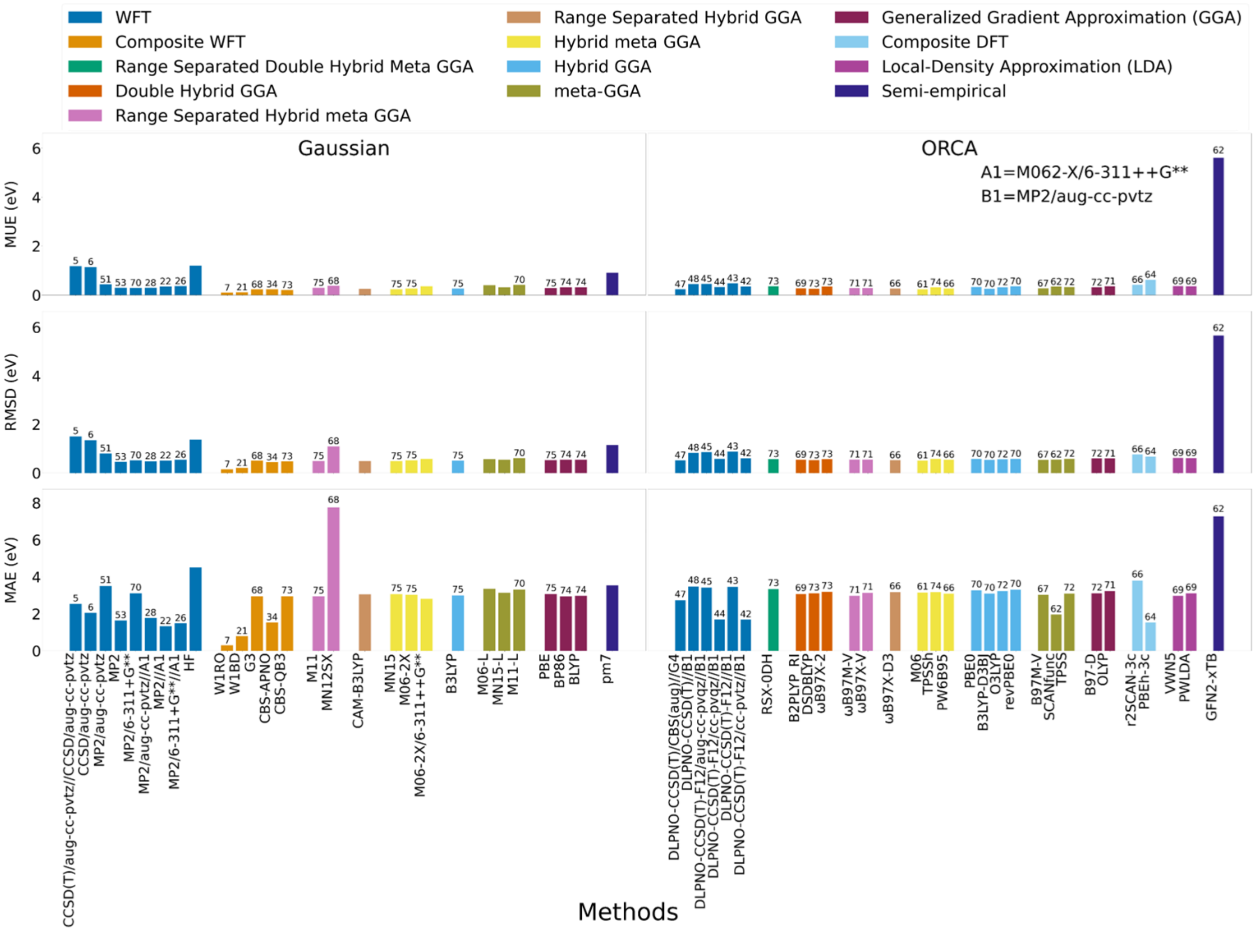

Figure S17: The MUE, RMSD, and max absolute error of electron affinity (EA) for all methods used in this study against G4 EA points (n=76) for neutral and neutral radical PFAS and PFAS-like molecules. For systems where certain EAs could not be obtained the corresponding value of n is indicated above the bar.

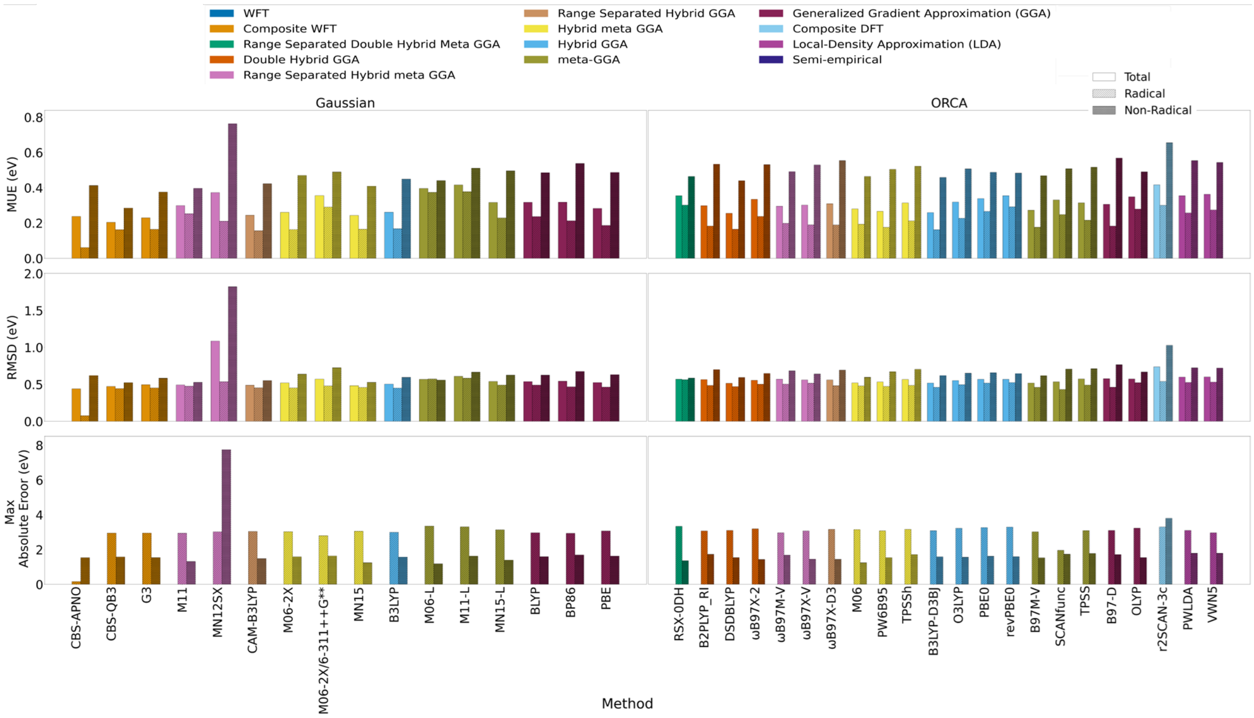

Figure S18: The MUE, RMSD, and max absolute error of electron affinity (EA) for all methods used in this study against G4 EA points split by neutral and neutral radical PFAS and PFAS-like molecules.

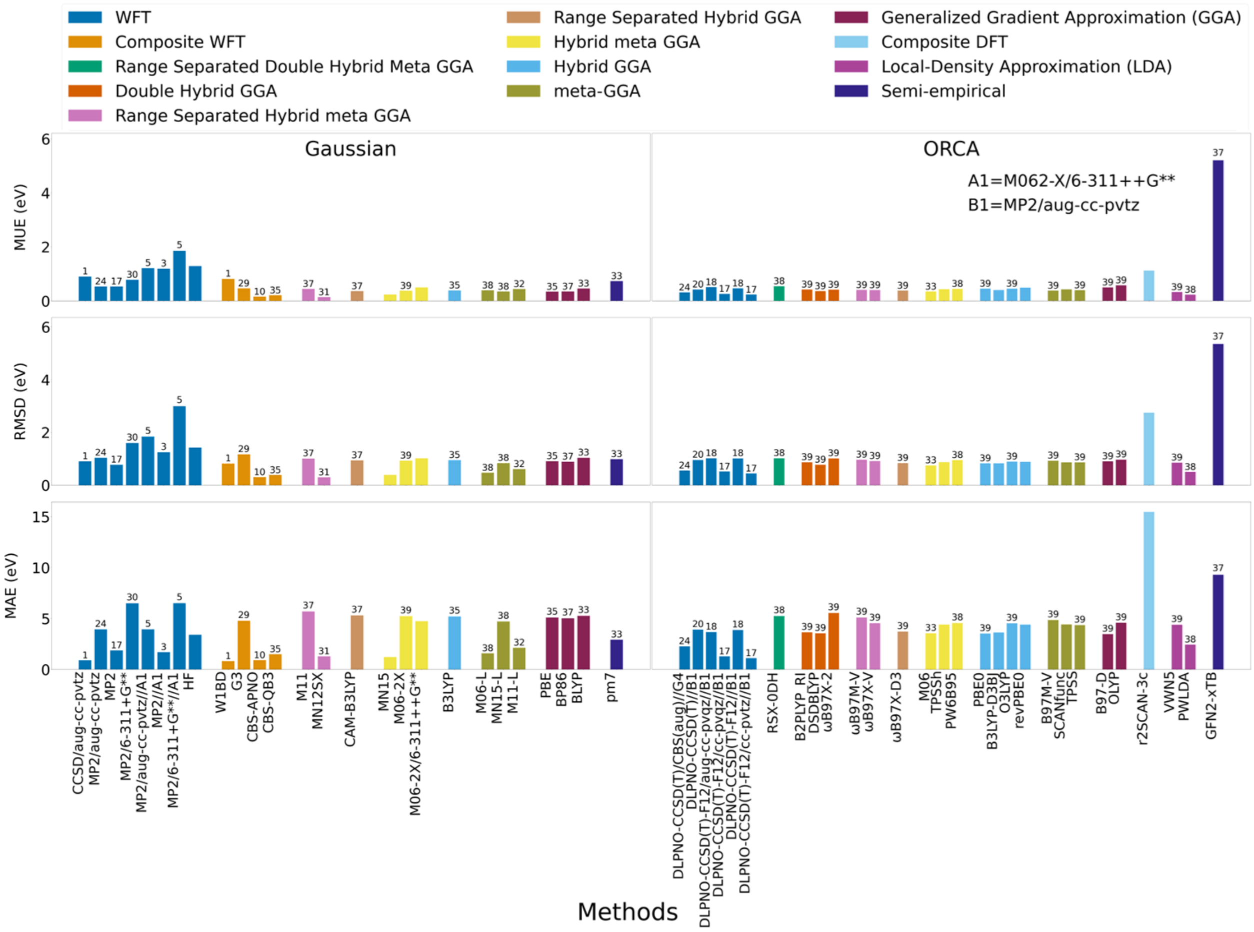

Figure S19: The MUE, RMSD, and max absolute error of electron affinity (EA) for all methods used in this study against G4 EA points (n=40) for anion and anion radical PFAS and PFAS-like molecules. For systems where certain EAs could not be obtained the corresponding value of n is indicated above the bar.
